# Asymmetrically Fluorinated Phenyl 4‐biphenylcarboxylate Motifs as a Design Principle for Enantiotropic Ferroelectric Liquid Crystals

**DOI:** 10.1002/advs.75740

**Published:** 2026-05-22

**Authors:** Hiroyuki Matsukizono, Koichiro Hayashi, Keiko Kojima, Yasushi Okumura, Hirotsugu Kikuchi

**Affiliations:** ^1^ Institute for Materials Chemistry and Engineering Kyushu University Kasuga Fukuoka Japan; ^2^ Interdisciplinary Graduate School of Engineering Science Kyushu University Kasuga Fukuoka Japan

**Keywords:** enantiotropic, ferroelectric liquid crystal, ferroelectric nematic, ferroelectric smectic

## Abstract

Understanding the molecular structures that exhibit ferroelectric liquid crystal (FLC) phases during both heating and cooling processes, i.e., enantiotropic FLC phases, remains a significant challenge. In this study, nine analogs based on a fluorinated phenyl 4‐biphenylcarboxylate backbone are designed, and their FLC properties are evaluated. Of these, eight analogs exhibit enantiotropic FLC phases. One analog containing the 2‐fluorobenzoate unit and a cyano terminal exhibits an enantiotropic ferroelectric nematic (N_F_) phase at 119°C–190°C. In contrast, analogs incorporating a 2,6‐difluorobenzoate unit show weakened or absent ferroelectric properties, whereas the introduction of a cyano terminal group leads to the emergence of an enantiotropic N_F_ phase. Quantum chemical calculations suggest that the planar orientation of the 2‐fluorobenzoate unit effectively promotes intermolecular interactions in parallel arrangements rather than antiparallel ones, thereby stabilizing FLC phases. Furthermore, an analog containing an asymmetrically fluorinated 4‐biphenylcarboxylate backbone exhibits an enantiotropic ferroelectric smectic A (SmA_F_) phase at 92°C–124°C. These results provide strong evidence that structural motifs comprising fluorinated biphenyl and planar benzoate skeletons are effective in promoting enantiotropic FLC phases. This study highlights the critical role of the mesogenic core structure (backbone) and provides valuable insights for the molecular design of enantiotropic FLC materials.

## Graphical abstract

The effect of the structural arrangement of DIO and its analogs on ferroelectric phases is explored. Consequently, it is found that an asymmetrically fluorinated phenyl 4‐biphenylcaroboxylate skeleton is one of the favorable structural motifs for the emergence of enantiotropic ferroelectric liquid crystal phases. The findings of our study provide valuable insights for the molecular design of materials showing enantiotropic ferroelectric nature.

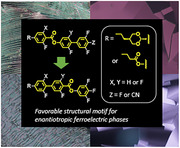

## 1 Introduction

Ferroelectric soft materials, which exhibit spontaneous polarization, are expected to find applications in a wide range of technologies. Among these materials, ferroelectric liquid crystals (FLCs) have attracted significant attention in recent years [1, 2]. Unlike conventional paraelectric liquid crystals (LCs), FLCs possess spontaneous polarization arising from broken symmetry. In the 1970s, research on FLC materials primarily focused on chiral smectic LCs [3, 4]; however, interest in FLCs expanded substantially after the nematic (N) phase of DIO was reported to exhibit ferroelectric behavior in 2017 [5]. In that report, the N phase—referred to as the MP phase—exhibited characteristic ferroelectric phenomena, including a large dielectric constant, polarization inversion, and second‐harmonic generation (SHG). In particular, the inversion of interference SHG fringes upon the reversal of the applied electric‐field polarity provided decisive evidence for the emergence of a ferroelectric nematic (N_F_) phase. In the same year as the DIO report, RM734 was reported to exhibit two types of N phases [6], and in 2020, the low‐temperature N phase was identified as the N_F_ phase [7]. Additionally, in 2021, UUQU‐4‐N was reported as the first compound exhibiting an N_F_ phase near room temperature [8]. Since the discovery of these FLCs, numerous analogs based on the corresponding molecular skeletons have been synthesized [9, 10, 11, 12, 13, 14, 15, 16, 17, 18, 19, 20, 21, 22, 23, 24, 25, 26, 27, 28, 29, 30, 31, 32, 33, 34, 35, 36, 37, 38, 39, 40, 41, 42, 43, 44, 45, 46]. To date, ferroelectric smectic A (SmA_F_) [9, 10, 11, 12, 13] and smectic C (SmC_F_) phases [13, 14, 15, 16], in which spontaneous polarization forms along the director, have been reported. More recently, FLC phases with macroscopic ordering, such as twist‐bend or heliconical N_F_ phases [13, 17] and heliconical polar smectic C phases [18] arising from broken chiral symmetry, have also been identified. The number of newly discovered FLCs is expected to continue increasing in the future, including reports of LCs whose ferroelectricity had previously been overlooked, such as RM734 and 3CN [19].

Although more than 300 FLCs have been reported to date, most exhibit monotropically appearing FLC phases that emerge only during supercooling below the melting point, and are therefore thermodynamically metastable. From an application perspective, materials that exhibit FLC phases during both heating and cooling, i.e., enantiotropic ferroelectric behavior, are highly desirable. However, identifying molecular structures that give rise to enantiotropic FLC phases remains a significant challenge, particularly because the fundamental mechanisms underlying FLC formation remain unclear. Currently, only a limited number of LCs have been reported to exhibit enantiotropic N_F_ [19, 20, 21, 22, 41, 42, 43, 44] and SmA_F_ phases [9, 10]. Moreover, these enantiotropic FLC phases typically occur over relatively narrow temperature ranges of 10–50°C. Rod‐shaped molecules reported by Nishikawa et al. exhibit enantiotropic N_F_ phases over substantially wider temperature ranges, reaching nearly 100°C depending on the alkyl chain length [17]. However, the enantiotropic N_F_ phase in these molecules still appears at relatively high temperatures, above 150°C. Consequently, the development of LCs that exhibit enantiotropic FLC phases at lower temperatures and across a wide temperature range remains a challenging issue.

To develop enantiotropic FLCs, it is essential to clarify the relationship between the molecular structure and ferroelectricity. Many studies have demonstrated that the emergence of FLC phases strongly depends on finely tuning parameters such as the magnitude and tilt angle of the dipole moment, molecular shape, and aspect ratio [23, 24]. Recently, the effects of fluorination on the emergence of FLC phases have been studied by several groups. Kikuchi et al. reported that the replacement of F atoms on the benzoate structure of DIO with H atoms favors the formation of SmA_F_ [9] and SmC_F_ phases [14] rather than the N_F_ phase. Gibb et al. emphasized the importance of specific fluorination patterns for stabilizing the N_F_ phase [21], while Strachan et al. demonstrated that introducing lateral F substituents can either suppress or promote the formation of ferroelectric smectic phases, depending on fluorination patterns [13]. In addition, the stability of FLC phases as a function of the fluorination pattern has been investigated by comparing electrostatic potential (ESP) surfaces based on Madhusudana's theoretical model [13, 21, 45, 47]. Despite these advances, the impact of structural sequences of main backbones on ferroelectricity is poorly discussed. In particular, there are no systematic reports evaluating the relationship between the molecular structure and enantiotropic FLC phases.

**FIGURE 1:**
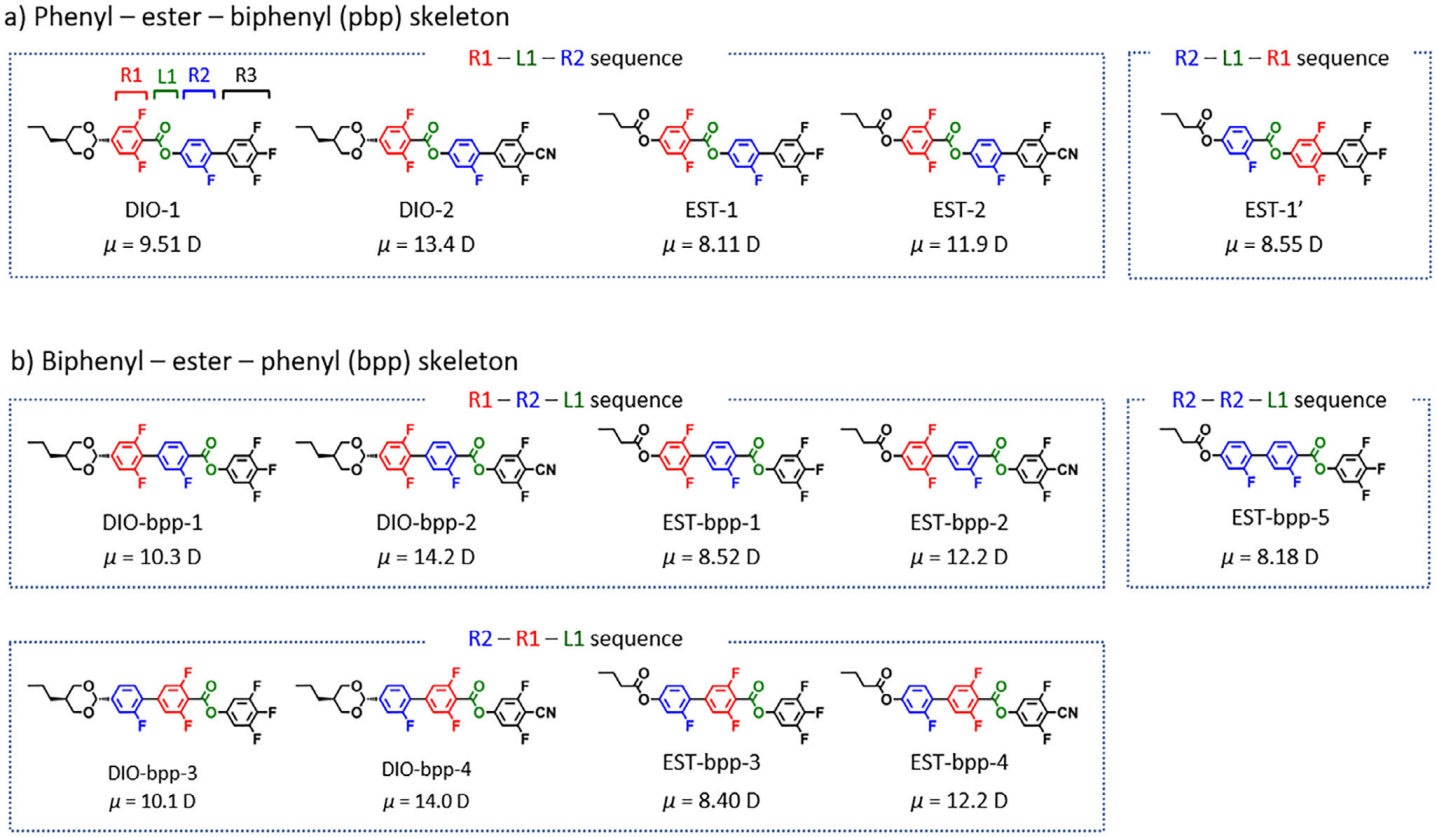
Chemical structures of DIO (**DIO‐1**) and its analogs composed of (a) phenyl–ester–phenyl (pbp) and (b) biphenyl–ester–phenyl (bpp) skeletons with the magnitudes of their dipole moments obtained by quantum chemical calculations. All analogs share common structural components: a difluorophenyl unit (R1), fluorophenyl unit (R2), an ester unit (L1), and phenyl terminals modified with F or CN groups (R3), while differing in the sequence of these components.

Many FLCs exhibiting enantiotropic characteristics incorporate benzoate and/or biphenyl units within their backbones [9, 10, 19, 20, 21, 22]. Accordingly, investigating the structural impact of these fundamental units on FLC phases is expected to provide valuable insights into the origins of ferroelectricity. In this study, we focused on the impact of these common structural elements on ferroelectricity and designed LC molecules with various arrangements of benzoate and biphenyl units. To facilitate clear structural comparisons with previously reported FLCs, we adopted simplified molecular architectures. As reference LCs, we selected DIO‐based analogs featuring a phenyl–ester–biphenyl (pbp) skeleton [5, 29] and DIO‐derived ester analogs in which the 1,3‐dioxane unit is replaced by an ester group (EST analogs) [10]. Based on these structures, we designed nine new analogs bearing a biphenyl‐ester‐phenyl (bpp) skeleton with different unit sequences (Figure 1). Remarkably, seven of these analogs were found to exhibit enantiotropic FLC phases. Here, we report a comprehensive evaluation of the FLC properties of these analogs and analyze the impact of their shared structural motifs on FLC phase formation. In addition, quantum chemical calculations were employed to elucidate the roles of the molecular structure and charge distribution in stabilizing enantiotropic FLC phases.

## 2 Results and Discussion

### 2.1 Structural Design and Dipole Moments of DIO and EST Analogs

**FIGURE 2:**
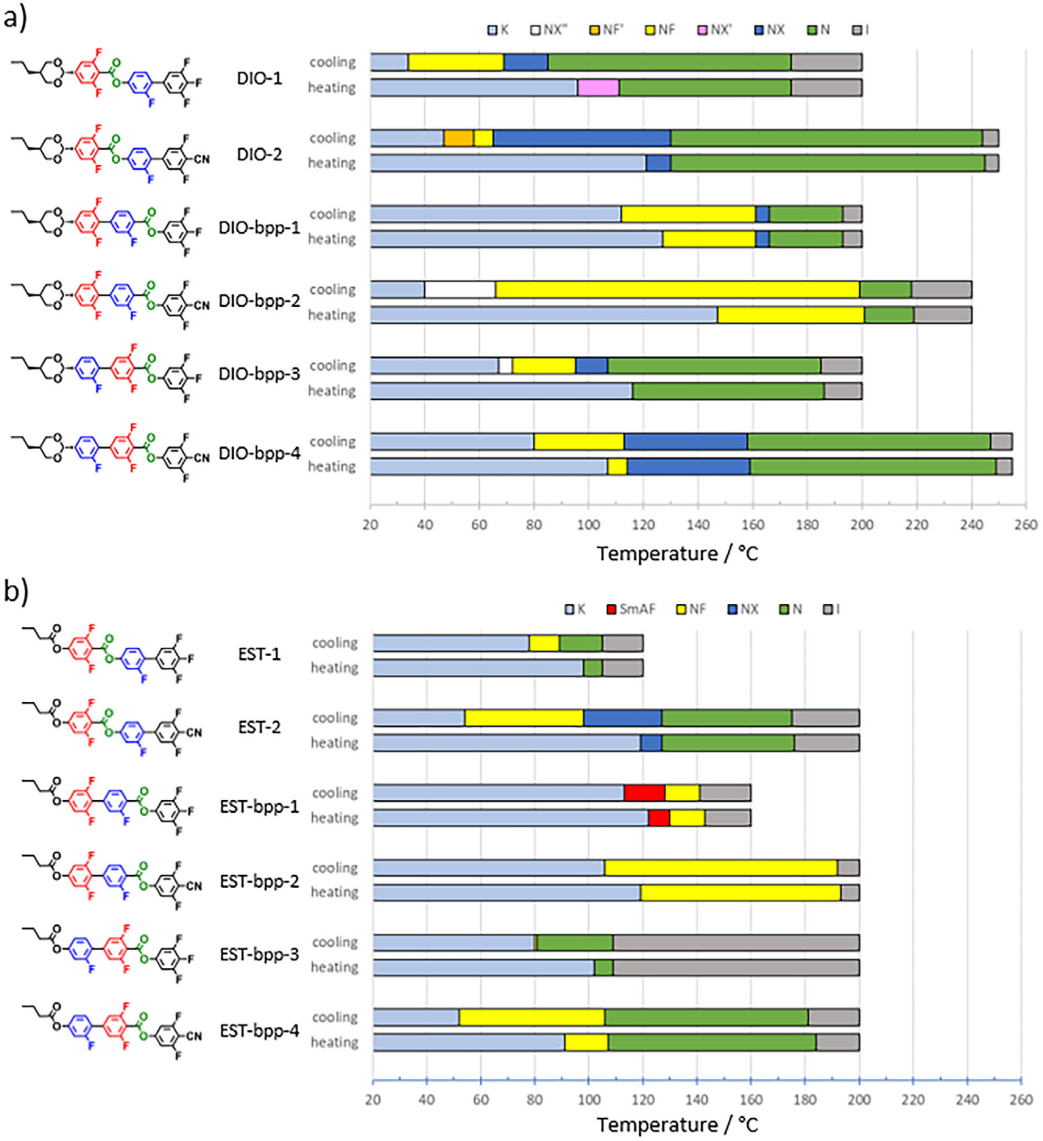
Summary of the phase‐transition characteristics of (a) DIO and (b) EST analogs during the heating and cooling processes. The phases were assigned based on the results of DSC, polarization reversal current profiles, and POM images. The abbreviations of phases are as follows: crystalline (K), nematic (N), isotropic (I), ferroelectric nematic (N_F_), ferroelectric smectic A (SmA_F_), other N (N_X_, N_X_’, and N_X_”), and another N_F_ (N_F_’). The N_X_ phase is determined to be the N phase with antiferroelectric nature (SmZ_A_ [34] and N_S_ [35]) by several studies. The N_X_’ phase is referred to as M3 phase in the original paper [5], which is different from the N and N_X_ phases. The N_F_’ phase is determined to be another N_F_ phase with weak ferroelectricity in the original paper [29]. For The N_X_” phase, the phase transition to this phase was only confirmed for POM observation.

Eight new DIO analogs (**DIO‐bpp‐n** and **EST‐bpp‐n**, *n* = 1–4) possessing the bpp skeleton were designed (Figure 1), comprising different sequences of difluorophenyl (R1), fluorophenyl (R2), and ester linkages (L1). **DIO‐bpp‐n** and **EST‐bpp‐n** (*n* = 1 and 2) have an R1–R2–L1 sequence, whereas **DIO‐bpp‐n** and **EST‐bpp‐n** (*n* = 3 and 4) adopt an R2–R1–L1 sequence. In addition, EST analogs with a pbp skeleton and an R2–L1–R1 sequence (**EST‐1‘**), as well as an EST analog with a bpp skeleton and an R2–R2–L1 sequence (**EST‐bpp‐5**), were also designed. These analogs were synthesized through multi‐step procedures, and their purity was verified by nuclear magnetic resonance (NMR) spectroscopy, high‐resolution mass spectrometry (HR‐MS), and elemental analysis (E.A.). The LC properties, including ferroelectricity, were primarily evaluated using differential scanning calorimetry (DSC), polarized optical microscopy (POM), and polarization reversal current measurements. Detailed results of the measurements and synthetic procedures are presented in Figures S1–S50. The phase transition behavior obtained from these results is shown in Figure 2 and Table S1.

The optimized structures and most stable molecular conformations of these analogs were determined by DFT calculations using the Gaussian16 software package [48]. The magnitudes of the dipole moments derived from these structures are shown in Figure 1. Structural optimization was performed using different basis sets (Figure S51 and Table S2). For **DIO‐bpp‐1**, the dipole moment obtained with the 6–31+G(2d,p) basis set (10.31 D) is nearly identical to that obtained with the aug‐cc‐pVTZ basis set; hence, the results based on the 6–31+G(2d,p) basis set were used for comparison. The dipole moments of **EST‐1** and **EST‐2** are slightly higher than those previously reported [10]. In contrast, the value for **DIO‐1** is slightly lower than that reported in other studies [8], which is attributed to differences in the basis sets used. Replacing the dioxane unit with an ester unit decreases the magnitude of the overall molecular dipole moment by approximately 1.5 D, whereas the introduction of a CN group increases it by approximately 3.8 D. DIO analogs with the R1–R2–L1 sequence show dipole moments approximately 0.8 D higher, while EST analogs with the same sequence exhibit increases of 0.3–0.4 D compared with those of their R1–L1–R2 counterparts. Analogs with the R2–R1–L1 sequence, in which the positions of the two phenyl units are interchanged, exhibit a slight decrease in their dipole moments compared with those of the original analogs with the R1–R2–L1 sequence. These results indicate that variations in the magnitude of the dipole moments are relatively minor among analogs with different biphenyl sequences.

### 2.2 Characterization of the Phase‐Transition Behavior

**FIGURE 3:**
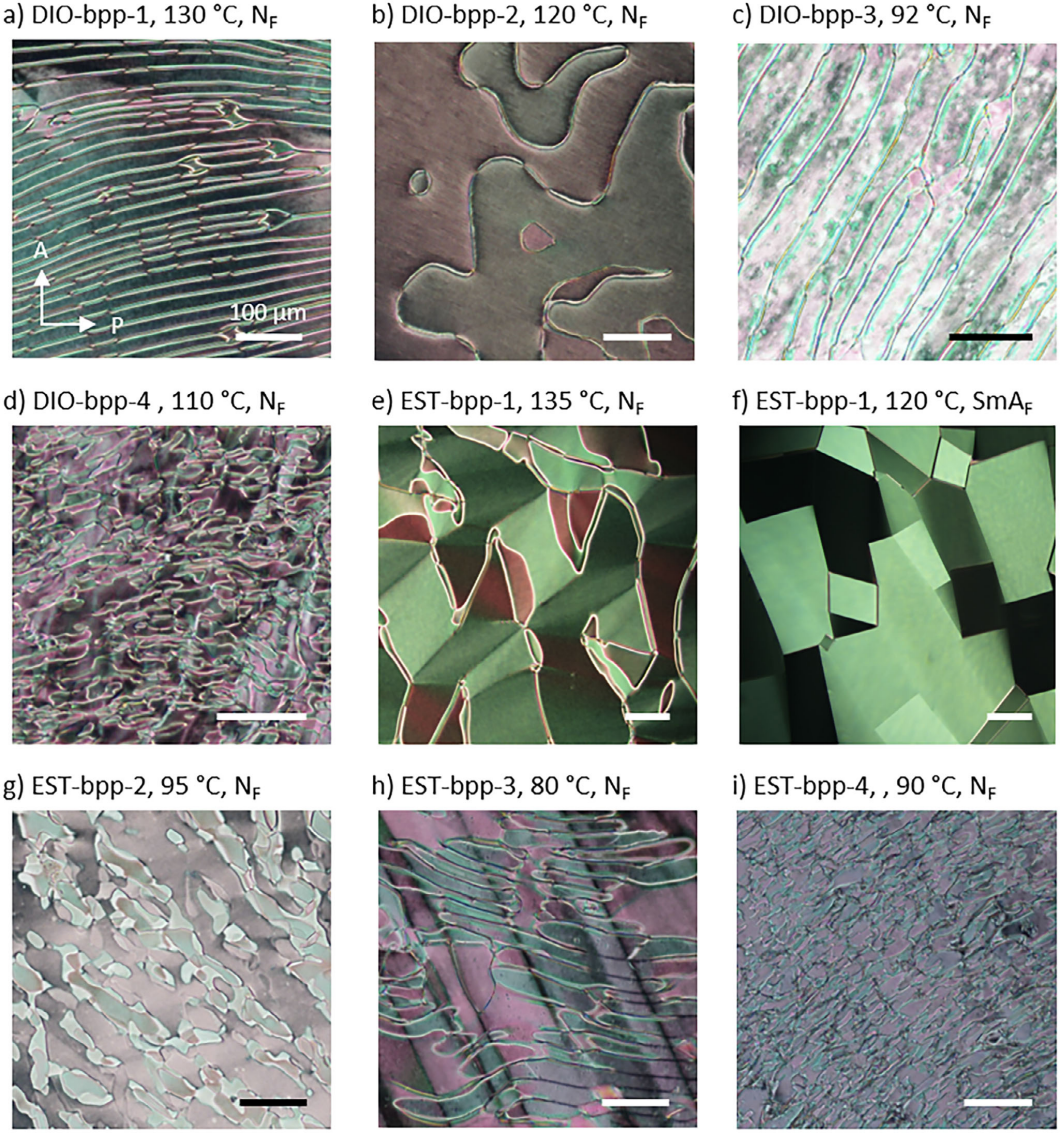
POM images of the ferroelectric liquid crystal (FLC) phases of (a–d) **DIO‐bpp‐n** and (e–i) **EST‐bpp‐n** (*n* = 1–4) during the cooling processes at a rate of 5 K min^−1^. Scale bar: 100 µm. Glass cells with non‐modified surfaces (gap: 10 µm) were used.

The POM images of **DIO‐bpp‐n** and **EST‐bpp‐n** (*n* = 1–4) recorded during the cooling process are shown in Figure 3. **DIO‐bpp‐1** and **DIO‐bpp‐3** exhibited textures with streaked disclination lines (referred to in the original paper [5] as “broken Schlieren textures”), which are characteristic of the N_F_ phase (Figure 3a,c). In contrast, **DIO‐bpp‐2**, **DIO‐bpp‐4**, **EST‐bpp‐1**, **EST‐bpp‐2**, and **EST‐bpp‐4** exhibited either fragmented stripe‐like textures or textures with two regions of uniform retardation (Figure 3b,d,e,g,i), both of which are also characteristic of the N_F_ phase. Meanwhile, **EST‐bpp‐3** showed a texture associated with the N_F_ phase only within a narrow temperature range immediately prior to crystallization (Figure 3h). Interestingly, only **EST‐bpp‐1** exhibited a large mosaic‐like texture with uniform retardation (Figure 3f), strongly suggesting the formation of the SmA_F_ phase [9, 10]. In contrast to the cooling process, the textures observed during heating were difficult to identify because of highly fragmented domains arising from the disordered molecular orientation during crystallization. With the exception of **DIO‐bpp‐3** and **EST‐bpp‐3**, the analogs are likely to display textures characteristic of the N_F_ phase.

**FIGURE 4:**
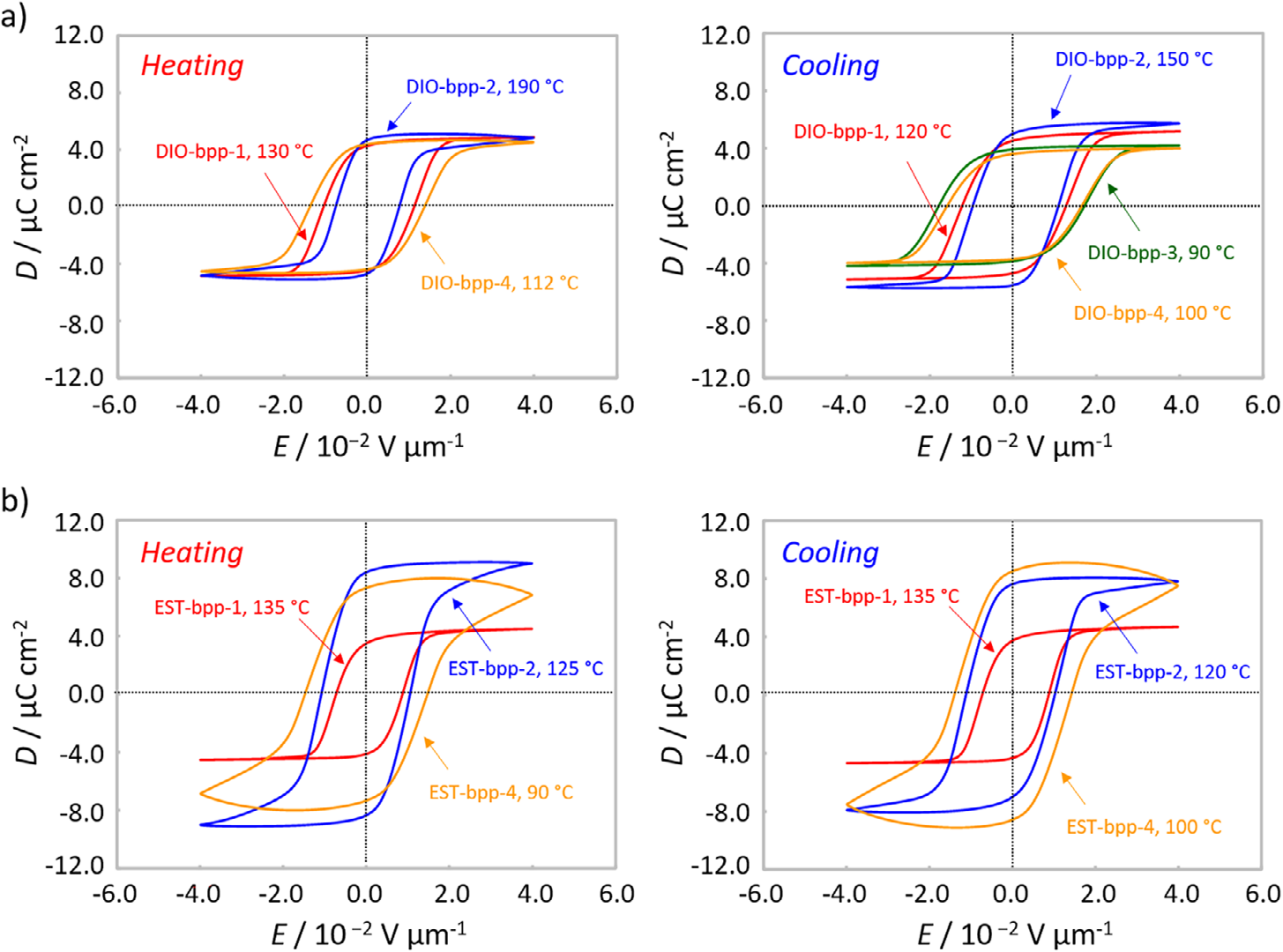
Current displacement–electric field (*D*–*E*) hysteresis loops of (a) **DIO‐bpp‐n** (*n* = 1–4) and (b) **EST‐bpp‐n** (*n* = 1, 2, and 4) recorded during the heating (left) and cooling (right) processes. For all compounds except **EST‐bpp‐3**, the profiles shown correspond to temperatures at which the ferroelectric nematic (N_F_) phase appears. Measurements were performed using an in‐plane switching cell (electrode area: 9 × 10^−4^ cm^2^; electrode distance: 500 µm). V_p–p_ = 40 V; frequency = 100 Hz.

To clearly evaluate these LC phases, polarization reversal currents were measured, and the corresponding *D*–*E* hysteresis loops were obtained (Figure 4). With the exception of **DIO‐bpp‐3** and **EST‐bpp‐3**, the analogs exhibited parallelogram‐shaped *D*–*E* hysteresis loops, clearly demonstrating ferroelectricity. Upon heating, the analogs with CN terminals displayed an abrupt increase in the current displacement within a narrow temperature range following their phase transitions, which may have been caused by the flow of a small amount of ionic impurities. Among these analogs, **EST‐bpp‐1** exhibited polarization reversal current peaks at 125°C, coinciding with the observation of the mosaic‐like texture. In the same temperature range, strong diffraction peaks assigned to the smectic layer spacing corresponding to its molecular length were confirmed by wide angle X‐ray diffraction (WAXD) measurements (Figure S15), indicating the emergence of the SmA_F_ phase. Based on these results, except for **DIO‐bpp‐3** and **EST‐bpp‐3**, the FLC phases observed in these analogs are enantiotropic LC phases that appeared during both the heating and cooling processes. These findings clearly demonstrate that the observed FLC phases are thermodynamically stable rather than metastable.

The detailed phase‐transition characteristics of these analogs are summarized in Figure 2. Among these analogs bearing the 1,3‐dioxane units, all analogs except **DIO‐bpp‐2** exhibited the N_X_ phase in the cooling process. The N–I transition temperatures of these analogs are higher than those of the corresponding EST analogs. In **DIO‐bpp‐2**, fine periodic micropatterns were generated within the domains during supercooling, as shown in the magnified image at 55°C in Figure S5. **DIO‐bpp‐3** exhibited a similar texture at 72°C (Figure S8). This phase might be the N_F_ phase, such as twist‐bend and heliconical N_F_ [13, 17], which tends to form polydomains to compensate for large spontaneous polarization. However, this phase was confirmed only by POM observation, and there are no other experimental data, including its ferroelectricity, because of the difficulty of analysis under supercooling. In this study, this phase is tentatively assigned to another N (N_X_″) phase. The temperature ranges in which the enantiotropic N_F_ phase forms are 34°C, 52°C, and 2°C for **DIO‐bpp‐1**, **DIO‐bpp‐2**, and **DIO‐bpp‐4**, respectively. The N_F_ phases of **DIO‐bpp‐1** and **DIO‐bpp‐2** were thermodynamically more stable than those of their original analogs. We next compared the EST series. Although both **EST‐1** and **EST‐2** exhibited the N_F_ phase, it appeared only during supercooling and over substantially wider temperature ranges. In contrast, **EST‐bpp‐1** and **EST‐bpp‐2** exhibited N_F_ phases over wider temperature ranges compared with those of their original analogs. Furthermore, **EST‐bpp‐1** exhibited an enantiotropic SmA_F_ phase. The temperature ranges of the enantiotropic FLC phases were 18°C and 15°C for **EST‐bpp‐1** and **EST‐bpp‐4**, respectively. Notably, **EST‐bpp‐2** exhibited the enantiotropic N_F_ phase over a temperature range of 71°C, which is an extraordinarily wide temperature range.

### 2.3 Evaluation of Structural Effects on FLC Phases

The impact of the substituents and structural sequences of these analogs on the emergence of FLC phases was evaluated. The EST‐bpp series exhibited a phase‐transition behavior similar to that of the corresponding DIO‐bpp series. In some cases, the EST‐bpp series showed simpler phase transitions and lower transition temperatures to the I phase. This tendency was consistent with that of the original DIO and EST series. Thus, the replacement of dioxane with ester units did not have a significant impact on the phase‐transition behavior. With a few exceptions, the introduction of the CN terminal tended to stabilize the FLC phases, which is attributed to an increase in the intermolecular dipole–dipole interactions along the longitudinal direction of the molecules. **DIO‐bpp‐2**, **DIO‐bpp‐4**, **EST‐bpp‐2**, and **EST‐bpp‐4**, which have large dipole moments above 12.0 D, exhibited the N_F_ phase. Only **EST‐bpp‐1** showed the SmA_F_ phase. In previous studies, EST analogs with the pbp skeleton exhibiting ferroelectric smectic phases did not possess strong electron‐withdrawing terminals [10]. However, there are a few molecules showing ferroelectric smectic phases, despite of the presence of CN and NO_2_ terminals [12, 46]. Although simple comparison is difficult due to the presence of dioxane moieties and multiple structural components, these results would suggest that molecules based on EST and EST‐bpp motifs without electron‐withdrawing terminals tend to be favorable for the emergence of ferroelectric smectic phases.

Next, we focused on the effects of the structural sequence, specifically the arrangement of rings or linkage units aligned in a single row along the molecular long axis for these analogs, excluding differences in the types of terminal substituents. The variation in the dipole moment resulting from the replacement of the ester bond and central phenyl unit, between the R1–L1–R2 and R1–R2–L1 sequences, is only 0.3–0.8 D, indicating that the significant differences in ferroelectricity are not due to dipole–dipole interactions. However, **DIO‐bpp‐1** exhibited the enantiotropic N_F_ phase, while **EST‐bpp‐1** exhibited both the enantiotropic N_F_ and SmA_F_ phases. Consequently, the **DIO‐bpp‐2** and **EST‐bpp‐2** analogs with the bpp skeleton, R1–R2–L1 sequence, and CN terminals exhibited the enantiotropic N_F_ phase over a remarkably wide temperature range exceeding 50°C. Interestingly, the positional change between the ester bond (L1) and central phenyl unit (R2) predominantly influenced the appearance of an enantiotropic N_F_ phase. These results provide useful insights for the molecular design of future N_F_ materials. In contrast, the exchange of the phenyl groups in the biphenyl unit, corresponding to the R2–R1–L1 sequence, drastically destabilized the FLC phases. **DIO‐bpp‐3** showed a monotropic N_F_ phase only during cooling, and the N_F_ phase was no longer stably present in **EST‐bpp‐3**. Among the analogs with biphenyl units, the largest structural difference was observed in the number of F atoms at the ortho position in the benzoate moiety. Thus, it is reasonable to consider that the steric hindrance around the benzoate moiety affects the appearance of ferroelectric phases. This effect is discussed in Section 2.4 based on DFT calculations. **DIO‐bpp‐4** and **EST‐bpp‐4**, which are analogs with CN groups, exhibited an N_F_ phase over narrow temperature ranges even during the heating process, despite having an R2–R1–L1 sequence. According to the Madhusudana model, the introduction of electron‐withdrawing terminals causes an increase in the surface charge density at the terminal, which may tend to destabilize the parallel arrangements. On the other hand, an increase in the dipole moment enhances dipole‐dipole interactions that can suppress thermal fluctuations, thus stabilizing the parallel arrangement of molecules [49]. In our system, it is predicted that the strong dipole‐dipole interactions mainly attributed the emergence of the N_F_ phase.

### 2.4 Comparison of Single Molecules After Structural Optimization

**FIGURE 5:**
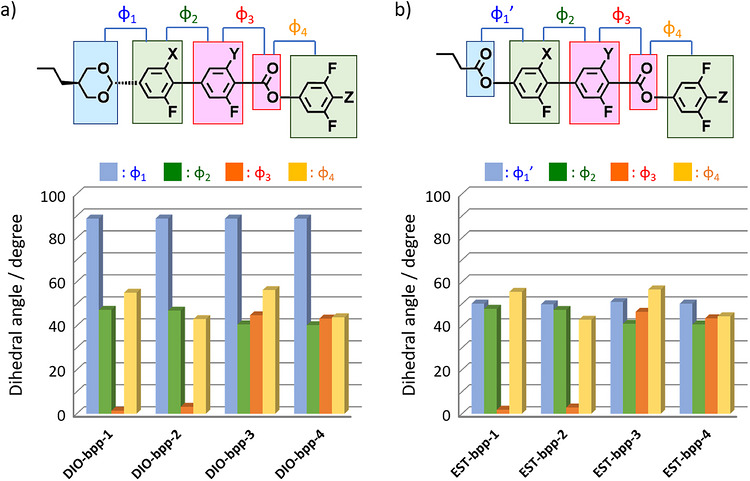
Comparison of the dihedral angles of optimized structures of (a) **DIO‐bpp‐n** and (b) **EST‐bpp‐n** (*n* = 1–4). X and Y = H or F; Z = F or CN. Optimized structures were determined by DFT calculations using the B3LYP/6‐31+G(2d,p) basis set. The dihedral angle between the 1,3‐dioxane unit and the adjacent benzene ring (φ_1_) is defined as the angle comprising the acetal hydrogen of 1,3‐dioxane and the adjacent carbons in the phenyl units. Other dihedral angles between the two phenyl groups in the biphenyl skeleton, carbonyl and phenyl groups in the benzoate unit, ester and terminal phenyl units, and carbonyl group in the butyryloxy skeleton and adjacent phenyl group are defined as, φ_2_, φ_3_, φ_4_, and φ_1'_, respectively.

To clarify the influence of the structural sequence of the molecule on the ferroelectricity, we compared the optimized molecular structures obtained from DFT calculations. Detailed structures are shown in Figure S51. The dihedral angles of the optimized structures are summarized in Figure 5. The dihedral angle between the dioxane and adjacent phenyl (φ_1_) units is defined as the angle between the acetal H atom of the dioxane unit and C atom of the neighboring phenyl unit. This angle is almost 90° for the DIO‐bpp series, indicating a planar arrangement of the dioxane and phenyl units. This result is consistent with the single‐crystal structure data of **DIO‐1** as well as with the calculated results of the analogs comprising the DIO skeleton. Conversely, the dihedral angles between the carbonyl groups in the butyryloxy unit and adjacent phenyl group (φ_1_’) are approximately 55° and remain constant across all EST‐bpp series. The dihedral angle between the benzene rings in the biphenyl unit (φ_2_) is approximately 40°–45°, and the angles in the biphenyl unit with the R1–R2–L1 sequence are slightly larger than those in the biphenyl unit with the R2–R1–L1 sequence. The dihedral angle between the ester and terminal phenyl (φ_4_) units is smaller than that between the ester unit and CN‐modified phenyl terminals. Although these differences are not large, a significant difference is observed in the dihedral angle between the carbonyl group and benzene ring in the benzoate unit (φ_3_). The dihedral angle is nearly 0° for the analogs with the R1–R2–L1 sequence consisting of a 2‐fluorobenzoate structure. In contrast, this angle is approximately 45° for the analogs with the R2–R1–L1 sequence comprising a 2,6‐difluorobenzoate structure. This difference is due to the steric repulsion between the two F atoms and the carbonyl oxygen.

Structural stability can be evaluated from the energy differences between the isomeric analogs (Table S3). The energy values of the analogs with the R1–R2–L1 sequence are larger than those of the analogs with the R2–R1–L1 sequence, and the energy difference is approximately 10 kJ mol^−1^, despite calculations using different basis sets. These results suggest that the planarity of the monofluorobenzoate moiety provides significant stabilization and contributes to the emergence of FLC phases.

Although **EST‐bpp‐1** and **EST‐bpp‐3** exhibit only a slight difference in the position of the F atom, they display significantly different ferroelectric properties. A detailed structural comparison of these analogs was performed using DFT calculations, and the relationship between the dihedral angle and energy is shown in Figure S52. These plots show the energy change of the optimized structures calculated after each dihedral angle was changed in approximate increments of 30° and then fixed (see the paragraph “Quantum chemical calculation” in Section S2). For φ_1_’, φ_2_, and φ_4_, the energy variations, Δ*E*, based on these changes are less than 3 kJ mol^−1^. In contrast, the change in φ_3_ significantly affected Δ*E*. For **EST‐bpp‐3**, the maximum Δ*E* was approximately 7 kJ mol^−1^ at 0° and 180°, whereas for **EST‐bpp‐1**, it reached an extremely large value of 16 kJ mol^−1^ at 90° and 270°. The maximum Δ*E* in **EST‐bpp‐1** was approximately 2.3 times that of **EST‐bpp‐3**. These results indicate that the planarity of the benzoic acid moiety is favored when the phenyl group adjacent to the ester linkage L1 is R2 (fluorophenyl) rather than R1 (difluorophenyl). Because of this effect, **EST‐bpp‐1** possesses a structure that is advantageous for maintaining the planarity of the mesogenic core of the molecule.

### 2.5 Comparison of Optimized Structures After Pairing in Parallel/Antiparallel Arrangements

**FIGURE 6:**
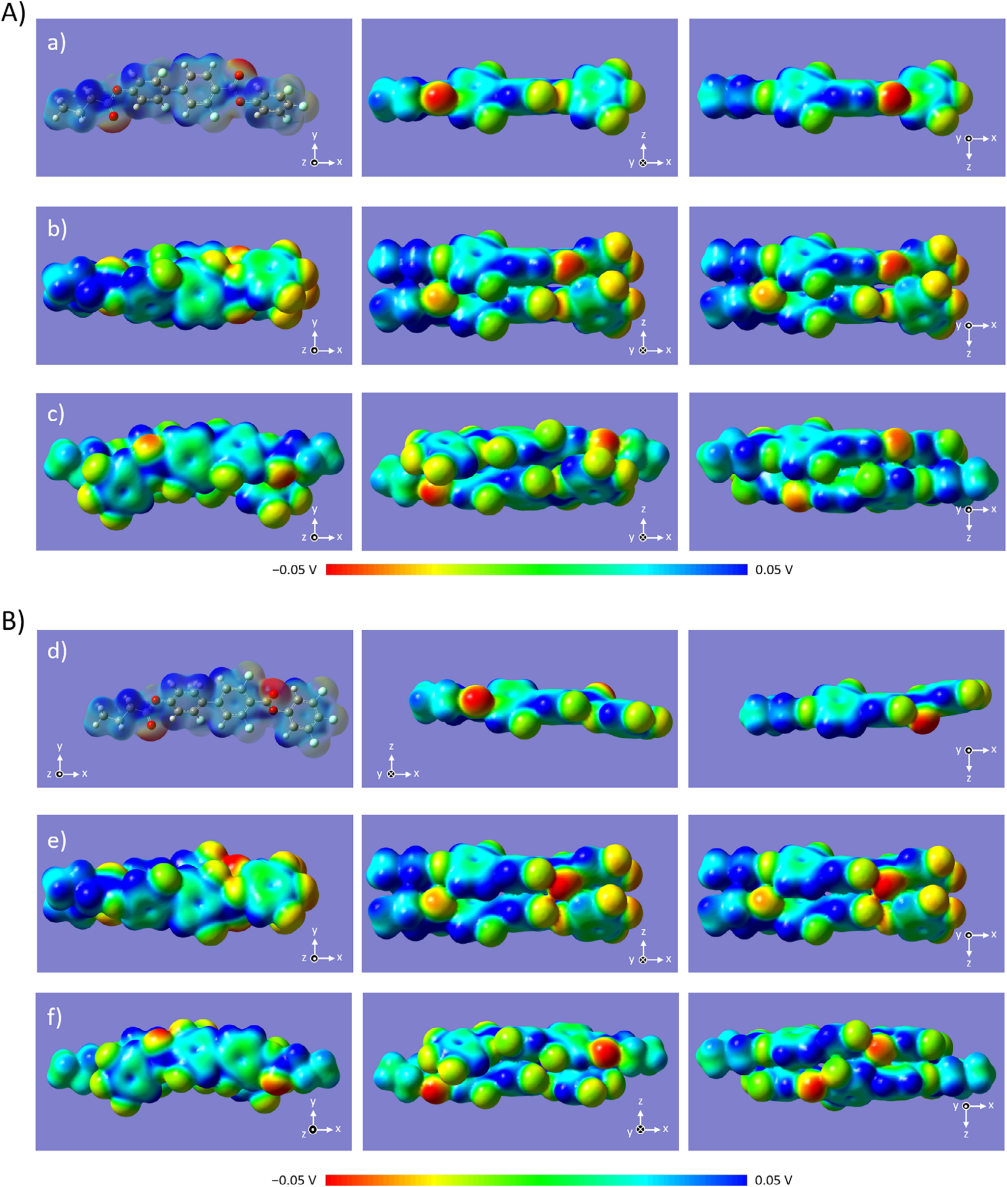
Comparison of the electrostatic potential (ESP) surfaces for (A) **EST‐bpp‐1** and (B) **EST‐bpp‐3**: (a,d) single molecules; (b,e) pairs of two molecules in parallel arrangement; and (c, f) pairs of two molecules in antiparallel arrangement. Optimized structures were obtained by DFT calculations using the B3LYP/6‐31+G(d,p)/GD3BJ basis set. The ESP surfaces for the pairing patterns with the largest stabilization energy are shown. The details for the pairing are summarized in Figures S53–S55 and Tables S4 and S5 in the ESI section. The electrostatic potential surfaces are mapped onto the electron density of the optimized structures with an iso‐value level of 0.004.

To evaluate how structural differences affect the conformation of an entire molecule, it is important to consider intermolecular interactions because of the collective nature of their LC phases. As the simplest case, we focused on the structures formed by two molecules in close proximity, calculated their optimized structures, and compared their conformational, energy, and dipole‐moment properties. Similar computational comparison between parallel and antiparallel arrangements has already been studied, by which the stabilization energy of RM734 series and molecules with a pbp skeleton were quantitatively evaluated [21, 33]. For parallel and antiparallel arrangements of two single molecules, optimized structures of several pairing patterns of **EST‐bpp‐1** and **EST‐bpp‐3** were calculated, and their stabilization energies were compared (Figures S53–S55 and Table S4). In these figures and tables, the parallel and antiparallel pairing patterns with the largest stabilization energy are exhibited in red and blue, respectively. Figure 6 shows the electrostatic potential (ESP) surfaces mapped onto the electron densities of the optimized structures for single molecules and their pairs arranged in parallel and antiparallel with the largest stabilization energy. For single molecules, the carbonyl oxygen atoms in the ester linkages carry a strong negative charge, and the F atoms exhibit a weak negative charge, whereas the surrounding H and C atoms are strongly positively charged (Figure 6a,d). The longitudinal ends are relatively weakly charged. However, in the parallel pair of the **EST‐bpp‐1** molecules, the negative charge of the carbonyl oxygen in the butyryloxy group is weakened. Another carbonyl oxygen atom remains negatively charged, and its charge is effectively stabilized by the surrounding positive charges (Figure 6b). These results suggest that the parallel arrangement of the molecules affords electrostatic stabilization. Interestingly, the positive/negative charges at the terminal parts along the long axis are slightly stronger than those in a single molecule. This charge distribution presumably promotes molecular orientation along the long axis, which is favorable for the appearance of FLC phases. The dipole moment of 17.0 D for the parallel pair, which is approximately twice that of a single molecule, is sufficiently large and advantageous for the emergence of FLC phases. Meanwhile, the two carbonyl oxygen atoms carry strongly negative charges in the antiparallel pair (Figure 6c). In addition, the F atoms are slightly more negatively charged compared with the parallel pair. The positive/negative charges at the terminals along the long axis are effectively cancelled, and the dipole moment along the longitudinal axis decreases to 0.0 D, suggesting that the antiparallel pair favors the formation of paraelectric LC phases.

Similar to **EST‐bpp‐1**, in the parallel pair of **EST‐bpp‐3**, the negative charge of the carbonyl oxygen in the butyryloxy unit is weakened (Figure 6e). In contrast, the other carbonyl oxygen exhibits a relatively stronger negative charge, which slightly spreads to the neighboring F atom in the difluorobenzoate unit. For the antiparallel pair, the dipole moment along the longitudinal axis is nearly 0.0 D, indicating the formation of a desirable antiparallel arrangement. The carbonyl oxygens carry strong negative charges, and the charges at the terminals along the long axis are effectively neutralized (Figure 6f). These tendencies are similar to those observed for the antiparallel pair of **EST‐bpp‐1**. However, the F atoms carry relatively weak negative charges, suggesting the efficient stabilization of the antiparallel arrangement. Based on these results, we speculate that the parallel pair of **EST‐bpp‐1** is stabilized more effectively than that of the antiparallel pair, whereas the difference in stability between the parallel and antiparallel pairs of **EST‐bpp‐3** is relatively small.

Next, we compared the energy differences between an isolated single molecule and a pair of molecules in close proximity. The corrected stabilization energy of **EST‐bpp‐1** for the formation of the parallel pair from two isolated molecules is calculated to be −113.6 kJ mol^−1^ (Table S5). The stabilization energy between the antiparallel pair and two isolated molecules is calculated to be −97.1 kJ mol^−1^, indicating that the antiparallel pair is 16.5 kJ mol^−1^, less stable than the parallel pair. On the other hand, the stabilization energies of the parallel and antiparallel pairs of **EST‐bpp‐3** are calculated to be −100.1 and −98.5 kJ mol^−1^, respectively (Table S5). The energy difference of 1.6 kJ mol^−1^ is one‐10th smaller than that of **EST‐bpp‐1**. These values of stabilization energy differences between the parallel and antiparallel arrangements are approximately comparable to those reported for RM734 (−26.8 kJ mol^−1^) [33]. Based on these results, we concluded that the parallel arrangement of **EST‐bpp‐3** is less stable than that of **EST‐bpp‐1**, making **EST‐bpp‐3** more prone to exchange between the parallel and antiparallel configurations, reducing the stability of the FLC phases. Notably, the energy difference between the parallel and antiparallel pairs of **EST‐bpp‐1** is 14.9 kJ mol^−1^ larger than that of **EST‐bpp‐3**, which is comparable to the energy barriers associated with the conformational changes between the phenyl and carbonyl groups in the 2‐fluorobenzoate unit of **EST‐bpp‐1** (Figure S52b). Although this energy difference reflects a single‐molecule interaction, these effects accumulate within molecular assemblies with LC properties, and are therefore expected to yield a greater stabilizing effect at the mesoscopic scale.

### 2.6 Comparison of Structural Arrangements in Molecular Backbones

Finally, we evaluated the structural impact of the bpp skeleton on the phase‐transition behavior by comparison with the pbp skeleton. Analogs bearing the bpp skeleton with an R1–R2–L1 sequence exhibit higher melting points and phase‐transition temperatures than those bearing the pbp skeleton with an R1‐L1‐R2 sequence. In contrast, analogs of the bpp skeleton with an R2–R1–L1 sequence show melting points and phase‐transition temperatures comparable to those of the pbp skeleton with an R1–L1–R2 sequence. Because the R1–L1 sequence is common in these analogs, these results indicate that the 2,6‐difluorobenzoate unit strongly influences the lowering of phase‐transition temperatures. Furthermore, analogs possessing the pbp skeleton tend to exhibit monotropic FLC phases, although exceptions have been reported [9, 10, 21]. In contrast, it is noteworthy that analogs with the bpp skeleton preferentially exhibit enantiotropic FLC phases.

**FIGURE 7:**
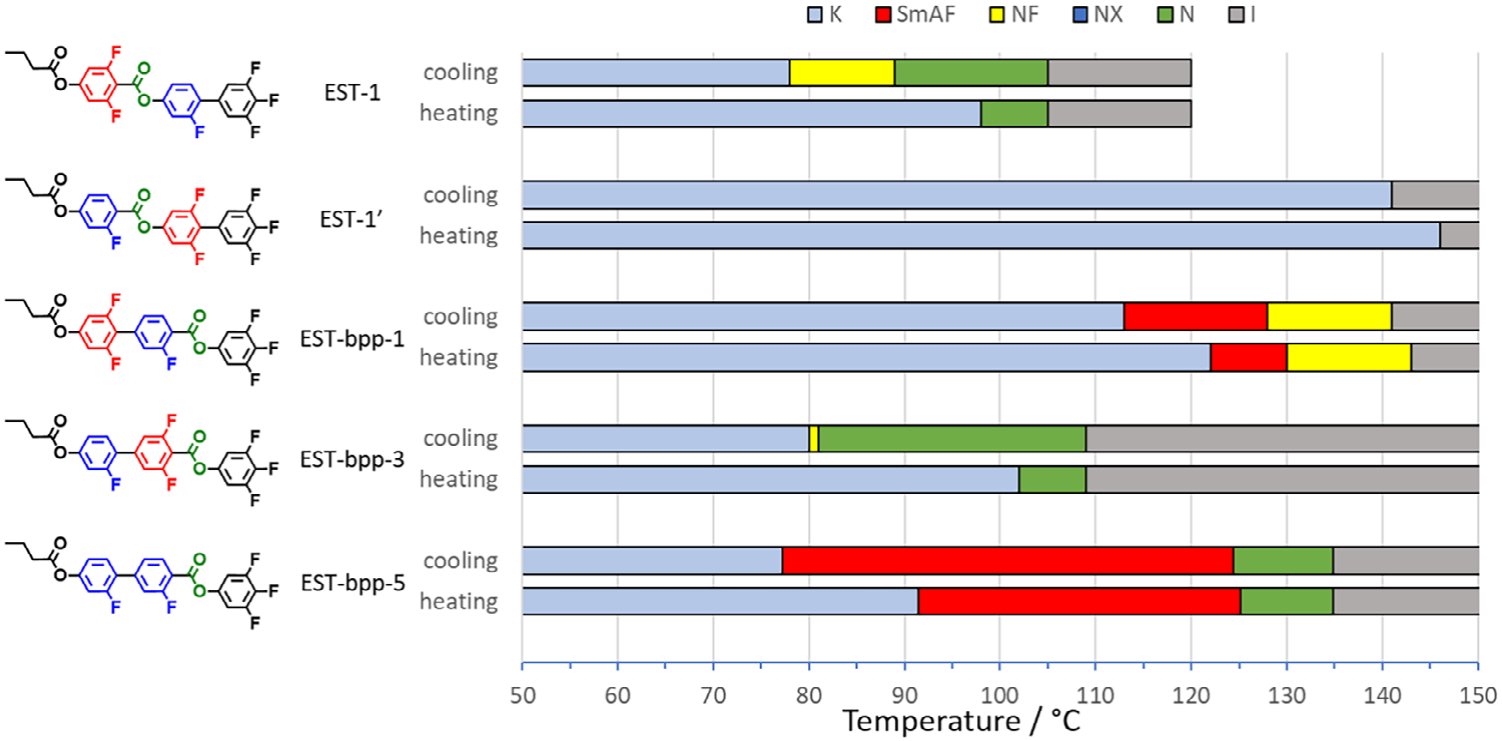
Comparison of the phase‐transition characteristics of EST and EST‐bpp analogs composed of different structural sequences during the heating and cooling processes. The phases were assigned based on the results of DSC, polarization reversal current profiles, and POM images.

To examine the effect of structural sequences in detail, **EST‐1’**, which comprises the pbp skeleton with an R2–L1–R1 sequence, was synthesized, and its LC properties were investigated (Figure 7). Only a K–I phase transition was observed at 147°C upon heating and 140°C upon cooling (Figures S26 and S27). As a result, no LC phases appeared, indicating that molecules with a pbp skeleton and an R2–L1–R1 sequence are unfavorable for the emergence of FLC phases. However, FLC phases have been reported for LCs with a pbp skeleton and an R2–L1–R1 sequence [13, 21]. Additionally, there is a report that DIO analogs with a pbp skeleton and a R1‐L1‐R1 sequence stabilizes the N_F_ phase despite the increase in structural symmetry [44]. A direct comparison would not be appropriate because the reported LCs are composed of complicated structures or include a CN terminal, dioxane, and 2,6‐difluorobenzoate units.

The higher melting point of **EST‐1’** (above 140°C) is attributed to the higher symmetry of the fluorinated biphenyl moiety, suggesting that lowering the molecular symmetry may promote the formation of LC phases. To test this hypothesis, we designed **EST‐bpp‐5**, consisting of a lower‐symmetry structure with an R2–R2–L1 sequence, and investigated its FLC properties (Figures S28–S30). The results are shown in Figure 7. In this compound, the N_F_ phase disappeared, and an N phase appeared instead. The destabilization of the N_F_ phase caused by the reduction of F atoms at the inner positions of the biphenyl unit is consistent with previous reports [13, 21]. Notably, however, **EST‐bpp‐5** exhibits an enantiotropic SmA_F_ phase over a significantly wider temperature range. Although the upper temperature limit is slightly reduced, the lower limit decreases markedly to 92°C, consequently extending the temperature range over 30°C. To the best of our knowledge, this is the first report of LCs exhibiting an enantiotropic SmA_F_ phase at temperatures below 100°C. This stabilization of the enantiotropic SmA_F_ phase has not been reported previously and contradicts previous reports suggesting that the reduction of F atoms in the biphenyl unit destabilizes the ferroelectric smectic phase [13]. Unlike previously reported LCs, which do not show any substantial reductions in the melting point, **EST‐bpp‐5** exhibits a melting point approximately 30°C lower than that of **EST‐bpp‐1**. This result suggests that the asymmetric R2–R2 biphenyl structure weakens molecular packing, destabilizes the crystalline phase, and thereby expands the temperature range over which the enantiotropic SmA_F_ phase appears. Collectively, these results provide strong evidence that a structural motif combining an asymmetrically fluorinated biphenyl unit with a benzoate moiety constitutes a favorable framework for the emergence of enantiotropic FLC phases.

## 3 Conclusion

In this study, we synthesized ten DIO‐based analogs comprising pbp and bpp skeletons with different sequences of F‐substituted phenyl units and ester linkers, and evaluated the correlation between their molecular structure and LC properties by comparing their phase‐transition behavior and DFT calculations. By POM observation, DSC, polarization reversal current, and WAXD measurements, it is revealed that these analogs exhibit ferroelectric LC phases as well as conventional para‐ and antiferroelectric LC phases. Additionally, a transition from the N_F_ phase to another phase with periodic structures appeared for **DIO‐bpp‐2** and **DIO‐bpp‐3** upon supercooling, which is tentatively assigned to N_X_″ phase in this study. The R1–R2–L1 sequence of the bpp skeleton favored the emergence of FLC phases, while the R2–R1–L1 sequence hindered the formation of FLC phases. A comparison of the optimized structures revealed that the carbonyl and phenyl groups in the 2‐fluorobenzoate unit (the R2–L1 sequence) were arranged in a planar manner. In contrast, the carbonyl and phenyl groups of 2,6‐difluorobenzoate unit (the R1–L1 sequence) were twisted owing to steric hindrance. This difference affected the stability of the collective states formed by intermolecular interactions, such as LC phases. The influence of the R2‐L1 and R1‐L1 sequences on the stability of the LC phases has been observed in many molecules, and molecules with the 2‐fluorbenzoate unit tend to form more thermally‐stable LC phases [9, 13, 21, 22, 25], suggesting that this structural factor is common to a wide range of LC materials. To examine the simplest intermolecular interaction, we compared the optimized structures of two molecules arranged in parallel and antiparallel proximity to **EST‐bpp‐1** and **EST‐bpp‐3**. A comparison of the ESP surfaces suggested that the parallel arrangement of **EST‐bpp‐1** was stabilized more effectively than the antiparallel arrangement, while the difference in stability between the parallel and antiparallel arrangements of **EST‐bpp‐3** was relatively small. Furthermore, the energy difference between the parallel and antiparallel arrangements of **EST‐bpp‐1** was ten times greater than that of **EST‐bpp‐3**. Based on these results, we concluded that the analogs with the R2–R1–L1 sequence readily exchanged between parallel and antiparallel arrangements and thus formed a paraelectric phase. Conversely, the parallel arrangement was effectively stabilized in analogs with an R1–R2–L1 sequence, making these structures favorable for the emergence of thermodynamically stable FLC phases. By combining the R1–R2–L1 sequence with a CN terminal group, we successfully developed single‐component analogs that exhibit the enantiotropic N_F_ phase at 119°C–190°C. In addition, we found that **EST‐bpp‐5**, which possesses a lower‐symmetry bpp skeleton with an R2‐R2‐L1 sequence, exhibited an enantiotropic SmA_F_ phase at 92°C–124°C. To the best of our knowledge, this analog is the first example of an LC exhibiting an enantiotropic SmA_F_ phase at temperatures below 100°C with a temperature range exceeding 30°C. Taken together, these results suggest that structural motifs combining asymmetrically‐fluorinated biphenyl units with planar benzoate skeletons are particularly effective in promoting enantiotropic FLC phases. This study highlights the critical role of the molecular backbone and is expected to provide valuable guidance for the molecular design of FLC materials suitable for practical applications.

## Funding

JSPS KAKENHI Grant Numbers JP23H00303, JP23K04537, JP23K17366, JP23K26721.

## Conflicts of Interest

The authors declare no conflicts of interest.

## Supporting information




**Supporting File**: advs75740‐sup‐0001‐SuppMat.docx.

## Data Availability

The data that supports the findings of this study are available in the supplementary material of this article.
