# Supplementary material for: Asymmetrically Fluorinated Phenyl 4‐biphenylcarboxylate Motifs as a Design Principle for Enantiotropic Ferroelectric Liquid Crystals
Source: Adv Sci (Weinh). 2026 May 22;13(45):e75740. doi: 10.1002/advs.75740 (PMC13335911; doi:10.1002/advs.75740)
Supplement: Supplementary file 1 [file ADVS-13-e75740-s001.docx]

Supporting Information for

Asymmetrically fluorinated phenyl 4-biphenylcarboxylate motifs as a design principle for enantiotropic ferroelectric liquid crystals

Hiroyuki Matsukizono,*^a^ Koichiro Hayashi,^b^ Keiko Kojima,^a^ Yasushi Okumura,^a^ and Hirotsugu Kikuchi*^a^

^a^ Kyushu University, Institute for Materials Chemistry and Engineering, 816-8580, Japan

^b^ Kyushu University, Interdisciplinary Graduate School of Engineering Sciences, 816-8580, Japan

**1. Reagents**

Potassium carbonate (K_2_CO_3_), cesium carbonate (Cs_2_CO_3_), anhydrous sodium sulfate (Na_2_SO_4_), anhydrous magnesium sulfate (MgSO_4_), [tetrakis(triphenylphosphine)palladium(0)] ([Pd(Ph_3_)_4_]), dichloro [1,1’-bis(diphenylphosphino)ferrocene]palladium(II)] dichloromethane complex ([Pd(dppf)Cl_2_] DCM), potassium phosphate (K_3_PO_4_), *p*-toluenesulfonic acid monohydrate (TsOH·H_2_O), triethylamine (NEt_3_), pyridine and 1-ethyl-3-(3-dimethylaminopropyl)carbodiimide hydrochloride (WSC) were obtained from Wako Pure Chemical Co., Ltd. *n*-butyl lithium hexane solution (1.6 mol L^−1^), 4-dimethylaminopyridine (DMAP), and 10% palladium carbon (wetted with ca. 55% water) (Pd/C) were purchased from Tokyo Chemical Industry Co., Ltd. Fluorinated benzene derivatives and other common reagents including sodium hydroxide aqueous solution (NaOH aq.), hydrogen chloride aqueous solution (HCl aq.), methanol (MeOH), tetrahydrofuran (THF), N,N-dimethylformamide (DMF), ethyl acetate (EtOAc) and dichloromethane (DCM) were obtained from commercial sources. These reagents and solvents were used without any purification.

**2. Measurements**

*Measurements for syntheses*: NMR spectra were recorded on a JNM-ECZ400 spectrometer (JEOL Co., Ltd.) at 400 MHz for ^1^H NMR and 100 MHz for ^13^C{^1^H} NMR. Chemical shifts in NMR spectra are given in parts per million (ppm) downfield using tetramethylsilane as an internal standard. For ^19^F NMR spectroscopy, hexafluorobenzene was used as an internal standard. FT-IR spectroscopy was performed with a FT-IR spectrometer Spectrum Two (PerkinElmer Co., Ltd.) equipped with a MIRacle ATR apparatus (PIKE Technologies). The high-resolution mass spectroscopy (HRMS) was conducted on a JMS-700 instrument (JEOL Co., Ltd.) with a fast atom bombardment (FAB) mode using 3-nitrobenzyl alcohol as a matrix. Elementary analysis (E.A.) was performed by the Service Center of the Elementary Analysis of Organic Compounds, Faculty of Science, Kyushu University.

*Differential scanning calorimetry (DSC) measurements*: DSC profiles were recorded on a DSC 1 STARe System calorimeter (Mettler Toledo, Switzerland) with a dedicated aluminum pan. Heating and cooling processes were repeated 2–4 times at a scanning rate of 1–10 °C min^−1^. Temperature ranges of enantiotropic ferroelectric liquid crystal phases were determined by DSC profiles at 1st cycle.

*Polarized optical microscopy (POM) observations*: POM observation was conducted using Nikon ECLIPSE LV100NPOL with DS-Ri2 and DS-Ri3 cameras at a rate of 5 K min^−1^. The cells with non-treated glass substrates with a gap of 10 μm were used.

*Polarization reversal current measurements*: a waveform generator (2411B, Toyo Technica), an analog-to-digital converter (WaveBook 516A, Toyo Technica), and a current–voltage/charge–voltage (I–V/Q–V) converter (Model 6254C, Toyo Technica) were constructed. Samples were injected into a planar orientation ITO cell (EHC Co. Ltd., RIKE-2303) with an electrode length of 18 mm, a cell gap of 5 μm (cross-sectional area: 9 × 10^−4^ cm^2^) and an electrode distance of 500 µm. For **EST-bpp-5**, a homeotropic orientation cell (EHC Co. Ltd., KSHH-10/B111P6NSS05X) with a cell gap of 10 μm and an area of 1 cm^2^ was used. The polarization reversal current was measured using the triangular wave method at an applied voltage of ±20 – ±200 V and a frequency of 0.01–200 Hz. The electron displacement-electron field (*D*–*E*) hysteresis loops were obtained by integrating the measured currents.

*Wide angle X-ray diffraction (WAXD) measurements*: WAXD experiments of **EST-bpp-1** were performed at Kyushu University Beamline (SAGA-LS/BL06). Samples were poured into a hole of the stage (diameter = 3 mm, depth = 1 mm) equipped with a pair of magnets (magnetic field = approximately 560 mT). The stage was set at which the direction of magnetic field (the direction of the director of molecules) aligned perpendicular to the direction of X-ray irradiation (detector distance = 179 mm, wavelength = 0.20 nm). The measurement was performed in the cooling run at 145–90 °C with an aging time of 1 min and an exposure time of 10 sec. One-dimensional (1-D) profiles (2*θ* versus intensity plots) were obtained by the integration of the obtained 2D diffraction images.

*Quantum chemical calculation*: The optimized structures and dipole moment (*μ*) of single molecules were determined using DFT calculation with different basis sets in the Gaussian16 software program^[1]^. The absence of imaginary frequency was confirmed by the frequency calculation of each structure obtained. For **EST-bpp-1** and **EST-bpp-3**, the dependences of dihedral angles on the optimized structures were performed. After dihedral angle (φ1’) successively changed by approximately 30° from −180° to 180°, structural optimizations were carried out by DFT calculation with Gaussian16 software. In the condition, B3LYP/6-31+G(2d,p) basis set was adopted and used the opt=z-matrix keyword to fix the changed angle. For other dihedral angles (φ2–4), the same procedure was conducted. After the calculations, the energy differences from the lowest energy were plotted against the changes of dihedral angles, which are shown in Figure S52. The optimization calculation of pairs in parallel and antiparallel arrangements of **EST-bpp-1** and **EST-bpp-3** were performed using B3LYP/6-31+G(d,p)/GD3BJ basis set. The calculation for several pairs forming different contact faces were conducted and the structures with lowest energy are determined as the optimized structures after checking of no imaginary frequency. The energy values of the optimized structures of the pairs are corrected by the counterpoise (CP) method and stabilization energy were calculated by subtraction of the sum of two isolated molecules from the CP-corrected energy.

**3. Characterization**

**Table S1** Summary of phase transition properties including entropy changes.

^a^Transition temperature from K phase in the 1t heating process. ^b^Phase transition temperatures [°C]. Numbers in parentheses mean entropy changes (Δ*S*) [J mol^−1^ K^−1^]. Phases in parentheses denot the phases appeared in only POM observation. ^c^transition temperatures with Δ*S* from LC phases to the K phase.

3-1. **DIO-bpp-1**

**Figure S1** DSC charts of **DIO-bpp-1**. 1–2 cycles. Rate: 10 °C min^−1^.

**Figure S2** POM images of **DIO-bpp-1** during cooling (a–e) and heating processes (f–i). Scale bar: 100 μm. Glass cells with non-modified surfaces (gap: 10 μm) were used.

**Figure S3** Top: Polarization reversal current profiles of **DIO-bpp-1** under an applied triangular wave voltage during a) heating and b) cooling processes. Bottom: *D*-*E* hysteresis loops during c) heating and d) cooling processes. V_P–P_ = 40 V. *f* = 100 Hz.

3-2. **DIO-bpp-2**

**Figure S4** DSC charts of **DIO-bpp-2**. 1–2 cycles. Rate: 10 °C min^−1^.

**Figure S5** POM images of **DIO-bpp-2** during cooling (a–f) and heating processes (g–i). Scale bar: 100 μm. Glass cells with non-modified surfaces (gap: 10 μm) were used.

**Figure S6** Top: Polarization reversal current profiles of **DIO-bpp-2** under an applied triangular wave voltage during a) heating and b) cooling processes. Bottom: *D*-*E* hysteresis loops during c) heating and d) cooling processes. V_P–P_ = 40 V. *f* = 100 Hz.

3-3. **DIO-bpp-3**

**Figure S7** DSC charts of **DIO-bpp-3**. 1–2 cycles. Rate: 10 °C min^−1^.

**Figure S8** POM images of **DIO-bpp-3** during cooling (a–f) and heating processes (g–i). Scale bar: 100 μm. Glass cells with non-modified surfaces (gap: 10 μm) were used.

**Figure S9** Top: Polarization reversal current profiles of **DIO-bpp-3** under an applied triangular wave voltage during a) heating and b) cooling processes. Bottom: *D*-*E* hysteresis loops during c) heating and d) cooling processes. V_P–P_ = 40 V. *f* = 100 Hz.

3-4. **DIO-bpp-4**

**Figure S10** DSC charts of **DIO-bpp-4**. 1–3 cycles. Rates were 10 °C min^−1^ for 1st and 2nd cycles and 2 °C min^−1^ for 3rd cycle.

**Figure S11** POM images of **DIO-bpp-4** during cooling (a–e) and heating processes (f–i). Scale bar: 100 μm. Glass cells with non-modified surfaces (gap: 10 μm) were used.

**Figure S12** Top: Polarization reversal current profiles of **DIO-bpp-4** under an applied triangular wave voltage during a) heating and b) cooling processes. Bottom: *D*-*E* hysteresis loops in c) heating and d) cooling runs. V_P–P_ = 40 V. *f* = 100 Hz.

3-5. **EST-bpp-1**

**Figure S13** DSC charts of **EST-bpp-1**. 1–2 cycles. Rate: 10 °C min^−1^.

**Figure S14** POM images of **EST-bpp-1** during heating (a–c) and cooling processes (d–i). Scale bar: 100 μm. Glass cells with non-modified surfaces (gap: 10 μm) were used.

**Figure S15** a) 2D WAXD diffraction image and b) 1D profiles of **EST-bpp-1** at 120°C in the cooling run. λ = 0.20 nm. Exposure time = 10 sec. The sample was oriented along the direction of magnetic field (ca. 560 mT). The spots along the equatorial and meridional directions are assigned to the diffractions based on the orientations along long and short axes of the molecule, respectively. From the diffraction peaks, the *d*-spacing is determined to be 2.14 nm, which is well corresponded to the longitudinal length of the optimized structure (2.08 nm).

**Figure S16** Top: Polarization reversal current profiles of **EST-bpp-1** under an applied triangular wave voltage during a) heating and b) cooling processes. Middle: *D*-*E* hysteresis loops obtained from the above profiles during c) heating and d) cooling processes. V_P–P_ = 40 V. *f* = 100 Hz. Bottom: Polarization reversal current profiles of **EST-bpp-1** under an applied triangular wave voltage at 125 °C in e) heating and f) cooling runs. V_p-p_ = 200 V. *f* = 0.01 Hz.

3-6. **EST-bpp-2**

**Figure S17** DSC charts of **EST-bpp-2**. Rate: 10 °C min^−1^.

**Figure S18** POM images of **EST-bpp-2** during cooling (a–d) and heating processes (e, f). Scale bar: 100 μm. Glass cells with non-modified surfaces (gap: 10 μm) were used.

**Figure S19** Top: Polarization reversal current profiles of **EST-bpp-2** under an applied triangular wave voltage during a) heating and b) cooling processes. Bottom: *D*-*E* hysteresis loops obtained from the above profiles during c) heating and d) cooling processes. V_P–P_ = 40 V. *f* = 100 Hz.

3-7. **EST-bpp-3**

**Figure S20** DSC charts of **EST-bpp-3**. Rate: 10 °C min^−1^.

**Figure S21** POM images of **EST-bpp-3** during cooling (a–d) and heating processes (e, f). Scale bar: 100 μm. Glass cells with non-modified surfaces (gap: 10 μm) were used.

**Figure S22** Top: Polarization reversal current profiles of **EST-bpp-3** under an applied triangular wave voltage during a) heating and b) cooling processes. Bottom: *D*-*E* hysteresis loops obtained from the above profiles during c) heating and d) cooling processes. V_P–P_ = 40 V. *f* = 100 Hz.

3-8. **EST-bpp-4**

**Figure S23** DSC charts of **EST-bpp-4**. Rate: 5 °C min^−1^.

**Figure S24** POM images of **EST-bpp-4** during cooling (a–d) and heating processes (e, f). Scale bar: 100 μm. Glass cells with non-modified surfaces (gap: 10 μm) were used

**Figure S25** Top: Polarization reversal current profiles of **EST-bpp-4** under an applied triangular wave voltage during a) heating and b) cooling processes. Bottom: *D*-*E* hysteresis loops obtained from the above profiles during c) heating and d) cooling processes. V_P–P_ = 40 V. *f* = 100 Hz.

3-9. **EST-1’**

**Figure S26** DSC charts of **EST-1’**. Top: 1st sample, Rate: 5 °C min^−1^. Bottom: 2nd sample, Rate: 2 °C min^−1^.

**Figure S27** POM images of **EST-1’** during cooling and heating processes. Scale bar: 100 μm. Glass cells with non-modified surfaces (gap: 10 μm) were used

3-10. **EST-bpp-5**

**Figure S28** DSC charts of **EST-bpp-5**. Top: 1st cycle. Rates: 5 °C min^−1^. Bottom: 2nd cycle. Rates: 1 °C min^−1^.

**Figure S29** POM images of **EST-bpp-5** during cooling (a–d) and heating processes (e, f). Scale bar: 100 μm. Glass cells with non-modified surfaces (gap: 10 μm) were used

**Figure S30** Polarization reversal current profiles of **EST-bpp-5** under an applied triangular wave voltage during cooling processes. Top: Profiles at 130 °C. V_P–P_ = 40 V. *f* = 100 Hz. Bottom: Profiles at 110 °C. V_P–P_ = 140 V. *f* = 1 Hz.

**4. Synthesis**

4-1. Synthesis of 3,4,5-trifluorophenyl 4-{2,6-difluoro-4-(*trans*-5-n-propyl-1,3-doxoane-2-yl)phenyl}-2-fluorbenzoate (**DIO-bpp-1**)

4-1-1. 1-bromo-2,6-difluoro-4-(*trans*-5-n-propyl-1,3-doxoane-2-yl)benzene (**1**): To a DCM solution (50 mL) of 4-bromo-3,5-difluorobenzaldehyde (5.00 g, 22.6 mmol) and 2-n-propyl-propane-1,3-diol (2.97 g, 25.1 mmol) were added TsOH·H_2_O (475 mg, 2.50 mmol) and anhydrous MgSO_4_ (12.0 g, 99.7 mmol). The mixture was stirred at ca. 40 °C overnight and then, slowly added into distilled water (200 mL) at ambient temperature. After extraction by DCM (200 mL), the DCM layer was washed twice with distilled water (200 mL) and then, dried over anhydrous Na_2_SO_4_. After evaporation, the reside was purified by silica-gel column chromatography (hexane/EtOAc volume ratio of 50:1 to 10:1) to afford trans-isomer as colorless liquid in 3.62 g yield (49.9%). ^1^H NMR (400 MHz, CDCl_3_, ppm): δ 7.12–7.09 (m, 2H, Ar-H), 5,34 (s, 1H, acetal-H), 4.22 (dd, 2H, *J* = 11, 4.2 Hz, -CH_2_-O-), 3.51 (t, 2H, *J* = 11 Hz, -CH_2_-O-), 2.18-2.06 (m, 1H, C_3_H_7_-CH-), 1.38-1.29 (m, 2H, CH_3_-CH_2_-), 1.09 (q, 2H, *J* = 7.6 Hz, C_2_H_5_-CH_2_-), 0.93 (t, 3H, *J* = 7.4 Hz, CH_3_-). ^13^C{^1^H} NMR (100 MHz, CDCl_3_, ppm): δ 159.7 (dd, *J* = 247, 3.9 Hz), 140.5 (t, *J* = 8.2 Hz), 110.0–109.7 (m), 99.1, 97.9 (t, *J* = 24 Hz), 72.5, 33.8, 30.2, 19.5, 14.2. ^19^F NMR (376 MHz, CDCl_3_, ppm): δ −106.3 (2F, 2,6-difluorophenyl).

4-1-2. Methyl 4-{2,6-difluoro-4-(*trans*-5-n-propyl-1,3-doxoane-2-yl)phenyl}-2-fluorobenzoate (**2**): To a mixture of 1,4-dioxane (90 mL) and distilled water (10 mL) were added compound **1** (2.65 g, 8.25 mmol), 3-fluoro-4-methoxycarbonylphenylboronic acid (1.80 g, 9.09 mmol), Pd(dppf)Cl_2_ DCM (202 mg, 0.248 mmol, 3 mol%) and Cs_2_CO_3_ (8.08 g, 24.8 mmol). The mixture was stirred at 90 °C for 2 h in N_2_ atmosphere and then, solvents were removed under reduced pressure. The residue was added into distilled water (200 mL) and then, the mixture was neutralized by an addition of HCl solution. The solids were collected by suction filtration and then, purified by silica-gel column chromatography (hexane/DCM volume ratio of 1:1 to 0:1) followed by recrystallization from MeOH. Compound **2** was obtained as a white solid in 1.34 g yield (41.2%). ^1^H NMR (400 MHz, CDCl_3_, ppm): δ 8.01 (t, 1H, *J* = 8.0 Hz, Ar-H), 7.32-7.25 (m, 2H, Ar-H), 7.19-7.14 (m, 2H, Ar-H), 5.41 (s, 1H, acetal-H), 4.25 (dd, 2H, *J* = 12, 4.4 Hz, -CH_2_-O-), 3.96 (s, 3H, -COOCH_3_), 3.55 (t, 2H, *J* = 11 Hz, -CH_2_-O-), 2.21-2.09 (m, 1H, C_3_H_7_-CH-), 1.38-1.30 (m, 2H, CH_3_-CH_2_-), 1.10 (q, 2H, *J* = 7.7 Hz, C_2_H_5_-CH_2_-), 0.94 (t, 3H, *J* = 7.2 Hz, CH_3_-CH_2_-). ^13^C{^1^H} NMR (100 MHz, CDCl_3_, ppm): δ 164.6 (d, *J* = 2.6 Hz), 162.5 (d, *J* = 259 Hz), 159.6 (dd, *J* = 249, 6.7 Hz), 141.7 (t, *J* = 9.6 Hz), 135.7 (d, *J* = 9.6 Hz), 131.9, 126.0, 119.0 (d, *J* = 24 Hz), 118.1 (d, *J* = 9.5 Hz), 116.3 (t, *J* = 18 Hz), 110.0–109.7 (m), 99.3, 72.6, 52.5, 33.9, 30.3, 19.5, 14.2. ^19^F NMR (376 MHz, CDCl_3_, ppm): δ −110.8 (1F, 2-fluorobenzoate), −114.8 (2F, 2,6-difluorophenyl).

4-1-3. 4-{2,6-difluoro-4-(*trans*-5-n-propyl-1,3-doxoane-2-yl)phenyl}-2-fluorobenzoic acid (**3**): To a mixture of THF (12 mL) and MeOH (5 mL) were added compound **2** (1.34 g, 3.40 mmol), 5 M NaOH aq. (2 mL, 10 mmol) and distilled water (3 mL). The mixture was stirred at ambient temperature overnight to give a homogeneous solution. After evaporation, th residue was dispersed in distilled water (200 mL) and then, the pH of the solution was acidified to 3–4 by an addition of HCl solution. The precipitates were collected by suction filtration and washed thoroughly with distilled water. After drying under reduced pressure, a white solid was obtained in 1.16 g yield (89.9%). ^1^H NMR (400 MHz, DMSO-d_6_, ppm): δ 7.94 (t, 1H, *J* = 8.0 Hz, Ar-H), 7.46 (d, 1H, *J* = 11 Hz, Ar-H), 7.38 (d, 1H, *J* = 8.4 Hz, Ar-H), 7.25 (d, 2H, *J* = 8,4 Hz, Ar-H), 5.52 (s, 1H, acetal-H), 4.17 (dd, 2H, *J* = 12, 4.6 Hz, -CH_2_-O-), 3.55 (t, 2H, *J* = 11 Hz, -CH_2_-O-), 2.05-1.93 (m, 1H, C_3_H_7_-CH-), 1.34-1.25 (m, 2H, CH_3_-CH_2_-), 1.05 (q, 2H, *J* = 7.5 Hz, C_2_H_5_-CH_2_-), 0.88 (t, 3H, *J* = 7.2 Hz, CH_3_-). ^19^F NMR (376 MHz, DMSO-d_6_, ppm): δ −110.9 (1F, 2-fluorobenzoate), −114.3 (2F, 2,6-difluorophenyl).

4-1-4. **DIO-bpp-1:** To DCM (10 mL) containing compound **3** (761 mg, 2.00 mmol), 3,4,5-trifluorophenol (340 mg, 2.30 mmol) and DMAP (244 mg, 2.00 mmol) was added WSC (473 mg, 2.50 mmol). The mixture was stirred at ambient temperature for 3 h and then, added in DCM (40 mL). The DCM solution was washed with distilled water (50 mL) followed by ultra-pure water (50 mL × 2). After drying over anhydrous MgSO_4_, the DCM layer was concentrated, leaving a white solid in 1.2 g yield. The crude product was purified by silica-gel column chromatography (hexane/DCM volume ratio of 1:1 to 0:1) followed by recrystallization from acetone/MeOH to afford a white solid. The product obtained was further recrystallized from hexane/EtOAc to give **DIO-bpp-1** as a white solid in 650 mg yield (63.7%). ^1^H NMR (400 MHz, CDCl_3_, ppm): δ 8.13 (t, 1H, *J* = 7.4 Hz, phenyl), 7.40 (dd, 1H, *J* = 8,4, 1.2 Hz, Ar-H), 7.35 (dd, 1H, *J* = 12, 0.8 Hz, Ar-H), 7.22-7.17 (m, 2H, Ar-H), 7.02-6.94 (m, 2H, Ar-H), 5.42 (s, 1H, acetal-H), 4.26 (dd, 2H, *J* = 12, 4.4 Hz, -CH_2_-O-), 3.56 (t, 2H, *J* = 11 Hz, -CH_2_-O-), 2.21-2.10 (m, 1H, C_3_H_7_-CH-), 1.40-1.31 (m, 2H, C_2_H_5_-CH_2_-), 1.14-1.08 (m, 2H, CH_3_-CH_2_-), 0.94 (t, 3H, *J* = 7.4 Hz, CH_3_-). ^13^C{^1^H} NMR (100 MHz, CDCl_3_, ppm): δ 162.0 (d, *J* = 261 Hz), 161.7 (d, *J* = 3.8 Hz), 159.6 (dd, *J* = 250, 6.3 Hz), 151.2 (ddd, *J* = 250, 11, 5.0 Hz), 145.3 (td, *J* = 12, 4.0 Hz), 142.2 (t, *J* = 9.6 Hz), 139.6–137.2 (m), 137.3 (d, *J* = 9.5 Hz), 132.3, 126.4, 119.4 (d, *J* = 24 Hz), 116.6 (d, *J* = 9.6 Hz), 116.0 (t, *J* = 18 Hz), 110.0 (dd, *J* = 20, 6.7 Hz), 107.3 (dd, *J* = 17, 5.8 Hz), 99.3, 72.7, 34.0, 30.3, 19.6, 14.3. ^19^F NMR (376 MHz, CDCl_3_, ppm): δ −108.7 (1F, 2-fluorobenzoate), −114.8 (2F, 2,6-difluorophenyl), −133.8 (2F, 3,4,5-trifluorpphenyl), −164.3 (1F, 3,4,5-trifluorophenyl). HRMS (FAB+) m/z: calcd for C_26_H_20_F_6_O_4_: 510.1266; found: 511.1344 (M+H), Error: 0.0 ppm. Anal. Calcd for C_26_H_20_F_6_O_4_: C 61.18, H 3.95%; found: C 60.93, H 3.97%.

4-2. Synthesis of 4-cyano-3,5-diifluorophenyl 4-{2,6-difluoro-4-(*trans*-5-n-propyl-1,3-doxoane-2-yl)phenyl}-2-fluorbenzoate (**DIO-bpp-2**)

Compound **3** (400 mg, 1.05 mmol) and DMAP (122 mg, 1.00 mmol) were added in DCM (10 mL) and then, the mixture was stirred at ambient temperature. To the mixture, WSC (200 mg, 1.04 mmol) was added and then the mixture was stirred for ca. 10 min, resulting in a homogeneous solution. To the solution, 4-hydroxy-2,6-difluorobenzonitrile (162 mg, 1.04 mmol) was added and then the solution was stirred at rt. for 1h. After that, the solution was diluted by DCM (100 mL) and then washed with distilled water (100 mL) followed by ultra-pure water (100 mL × 2). After dried over anhydrous Na_2_SO_4_, the DCM layer was concentrated and then, the residue was purified by silica-gel column chromatography (hexane/DCM volume ratio of 1:3) followed by recrystallization from acetone/MeOH. The solid obtained was further purified by twice recrystallization by hexane/EtOAc. **DIO-bpp-2** was obtained as a white solid in 121 mg yield (27.0%). ^1^H NMR (400 MHz, CDCl_3_, ppm): δ 8.13 (t, 1H, *J* = 7.6 Hz, Ar-H), 7.42 (dd, 1H, *J* = 8.0, 1.5 Hz, Ar-H), 7.37 (d, 1H, *J* = 12 Hz, Ar-H), 7.22-7.18 (m, 2H, Ar-H), 7.12–7.09 (m, 2H, Ar-H), 5.42 (s, 1H, acetal-H), 4.26 (dd, 2H, *J* = 12, 4.6 Hz, -CH_2_-O-), 3.56 (t, 2H, *J* = 12 Hz, -CH_2_-O-), 2.21-2.10 (m, 1H, C_3_H_7_-CH-), 1.40-1.31 (m, 2H, CH_3_-CH_2_-), 1.14-1.08 (m, 2H, C_2_H_5_-CH_2_-), 0.94 (t, 3H, *J* = 7.4 Hz, CH_3_-). ^13^C{^1^H} NMR (100 MHz, CDCl_3_, ppm): δ 163.6 (dd, *J* = 261, 6.7 Hz), 162.0 (d, *J* = 264 Hz), 159.5 (dd, *J* = 249, 6.8 Hz), 159.6 (t, *J* = 13 Hz), 142.3 (t, *J* = 9.6 Hz), 137.8 (d, *J* = 9.5 Hz), 132.3, 126.5, 119.5, 115.9–115.6 (m), 110.0 (dd, *J* = 20, 6.8 Hz), 108.7, 107.0 (dd, *J* = 23, 3.8 Hz), 99.2, 90.3 (t, *J* = 19 Hz), 72.6, 33.9, 30.3, 19.5, 14.2. ^19^F NMR (376 MHz, CDCl_3_, ppm): δ −103.0 (2F, 4-cyano-3,5-difluorophenyl), −108.1 (1F, 2-fluorobenzoate), −114.8 (2F, 2,6-difluorophenyl). HRMS (FAB+) m/z: calcd for C_27_H_20_F_5_NO_4_: 517.1312; found: 518.1390 (M+H), Error: −0.1 ppm. Anal. Calcd for C_27_H_20_F_5_NO_4_: C 62.67, H 3.90, N 2.71%; found: C 62.71, H 3.93, N 2.66%.

4-3. Synthesis of 3,4,5-trifluorophenyl 2,6-difluor-4-{2-fluoro-4-(*trans*-5-n-propyl-1,3-doxoane-2-yl)phenyl}benzoate (**DIO-bpp-3**)

4-3-1. 1-bromo-2-fluoro-(5-n-propyl-1,3-dioxane-2-yl)benzene (**4**): To DCM (30 mL) were added 4-bromo-3-fluorobenzaldehyde (8.12 g, 40.0 mmol), 1,3-propanediol (5.20 g, 44.0 mmol) and TsOH H_2_O (836 mg, 4.40 mmol). To the solution, anhydrous MgSO_4_ (12.0 g, 100 mmol) was added and the mixture was stirred at 40 °C overnight. After cooling, the mixture was slowly poured into distilled water and then extracted with DCM. The organic layer was washed with distilled water and then dried over anhydrous MgSO_4_. After evaporation, a solid (12.1 g) was purified by silica-gel column chromatography (n-hexane/EtOAc volume ratio of 50:1 to 20:1) to afford compound **4** as a white solid in 7.25 g yield (59.8%). ^1^H NMR (400 MHz, CDCl_3_, ppm): δ 7.53 (dd, 1H, *J* = 8.4, 6.8 Hz, Ar-H), 7.28 (dd, 1H, *J* = 9.6, 2.0 Hz, Ar-H), 7.15 (dd, 1H, *J* = 8.4, 1.6 Hz, Ar-H), 5.46 (s, 0.1H, acetal-H in cis-form), 5.36 (s, 0.9H, acetal-H in trans-form), 4.23 (dd, 1.8H, *J* = 12, 4.8 Hz, -CH_2_-O- in trans-form), 4.10–4.03 (m, 0.4H, -CH_2_-O- in cis-form), 3.52 (t, 1.8H, *J* = 11 Hz, -CH_2_-O- in trans-form), 2.18–2.07 (m, 0.9H, C_3_H_7_-CH- in trans-form), 1.78–1.72 (m, 0.2H, CH_3_-CH_2_-CH_2_- in cis-form), 1.46–1.29 (m, 2.2H, CH_3_-(CH_2_)_2_- and C_3_H_7_-CH- in cis-form), 1.11–1.06 (m, 1.8H, CH_3_-CH_2_-CH_2_- in trans-form), 0.97–0.91 (m, 3H, CH_3_-). By ^1^H NMR spectroscopy, the cis/trans-isomer molar ratio of the solid was determined to be 1:9. The solid was used for the next step without further purification.

4-3-2. Methyl 2,6-difluoro-4-{2-fluoro-(trans-5-n-propyl-1,3-dioxane-2-yl)phenyl}benzoate (**5**): To a 1,4-dioxane (50 mL) of 3,5-difluoro-4-(methoxycarbonyl)phenylboronic acid pinacol ester (3.28 g, 11.0 mmol), compound **4** (3.03 g, 14.5 mmol), and [Pd(dppf)Cl_2_] (220 mg, 0.301 mmol, 3 mol%) were added Cs_2_CO_3_ (9.77 g, 30.0 mmol) and distilled water (5 mL). After stirred at 90 °C for 2 h under N_2_ atmosphere, the mixture was evaporated. The resulting solid was dissolved in EtOAc and filtrated to remove insoluble parts. The filtrate (150 mL) was washed three times with distilled water (150 mL), and then dried over anhydrous Na_2_SO_4_. After evaporation, the resulting brown solid was purified by silica-gel column chromatography (n-hexane/DCN volume ratio of 1:1 to 1:5), followed by recrystallization from acetone/MeOH to give compound **5** as a white solid in 2.62 g yield (66.5%). ^1^H NMR (400 MHz, CDCl_3_, ppm): δ 7.44–7.33 (m, 3H, Ar-H), 7.16 (dd, 2H, *J* = 8.0, 1.6 Hz, Ar-H), 5.44 (s, 1H, acetal-H in trans-form), 4.25 (dd, 2H, *J* = 12, 4.4 Hz, -CH_2_-O- in trans-form), 3.97 (s, 3H, -COOMe), 3.55 (t, 2H, *J* = 12 Hz, -CH_2_-O- in trans-form), 2.21–2.10 (m, 1H, C_3_H_7_-CH- in trans-form), 1.40–1.30 (m, 2H, CH_3_-CH_2_- in trans-form), 1.13–1.07 (m, 2H, C_2_H_5_-CH_2_- in trans-form), 0.94 (t, 3H, *J* = 7.4 Hz, CH_3_-CH_2_). ^19^F NMR (376 MHz, CDCl_3_, ppm): δ −111.2 (2F, 2,6-difluorobenzoate), −118.0 (1F, 2-fluorophenyl).

4-3-3. 2,6-difluoro-4-{2-fluoro-(trans-5-n-propyl-1,3-dioxane-2-yl)phenyl}benzoic acid (**6**): Compound **5** (2.37 g, 6.00 mmol) was dissolved in mixed solvents of THF (35 mL) and MeOH (10 mL). To the solution, 5 M NaOH aq. (3 mL, 15 mmol) was added, and then the mixture was stirred at rt. for 1.5 h. After evaporation, the residue was dispersed in H_2_O (150 mL), and then acidified by an addition of 1 M HCl aq. The resulting precipitates were collected by suction filtration and dried under reduced pressure to give compound **6** as a white solid in 2.02 g yield (88.6%). ^1^H NMR (400 MHz, DMSO-d_6_, ppm): δ 7.65 (t, 1H, *J* = 8.0 Hz, Ar-H), 7.44 (d, 2H, *J* = 8.8 Hz, Ar-H), 7.37–7.32 (m, 2H, Ar-H), 5.52 (s, 1H, acetal-H), 4.16 (dd, 2H, *J* 11, 4.4 = Hz, -CH_2_-O-), 3.54 (t, 2H, *J* = 11 Hz, -CH_2_-O-), 2.04–1.93 (m, 1H, C_3_H_7_-CH-), 1.34–1.25 (m, 2H, CH_3_-CH_2_-), 1.08–1.03 (m, 2H, C_2_H_5_-CH_2_-), 0.88 (t, 3H, *J* = 7.2 Hz, CH_3_-CH_2_). ^13^C{^1^H} NMR (100 MHz, DMSO-d_6_, ppm): δ 162.2, 159.4 (dd, *J* = 250, 7.7 Hz), 158.8 (d, *J* = 246 Hz), 142.2 (d, *J* = 8.7 Hz), 139.3 (t, *J* = 11 Hz), 130.8 (d, *J* =1.9 Hz), 125.5 (d, *J* = 3.8 Hz), 122.9 (d, *J* = 3.8 Hz), 114.1 (d, *J* = 24 Hz), 112.8 (d, *J* = 23 Hz), 111.9 (t, *J* = 20 Hz), 99.3, 71.8, 33.9, 30.0, 19.2, 14.4. ^19^F NMR (376 MHz, DMSO-d_6_, ppm): δ −112.2 (2F, 2,6-difluorobenzoate), −117.8 (1F, 2-fluorophenyl).

4-3-4. **DIO-bpp-3:** To DCM (25.4 mL) were added compound **6** (318 mg, 0.661 mmol), 3,4,5-trifluorophenol (108 mg, 0.727 mmol), DMAP (0.81 mg, 0.0066 mmol). To the mixture, WSC (139 mg, 0.727 mmol) was added and then, the mixture was stirred at rt. for 2 h. After that, WSC (59 mg, 0.31 mmol) was further added and then, the mixture was stirred at rt. overnight. The mixture was added in DCM and then washed with distilled water. The organic layer was dried over anhydrous MgSO_4_ and then concentrated. The residue was purified by silica-gel column chromatography (hexane/DCM volume ratio of 1/1) to afford **DIO-bpp-3** as a white slid. ^1^H NMR (400 MHz, CDCl_3_, ppm): δ 7.46-7.35 (m, 3H, Ar-H), 7.24 (d, 2H, *J* = 10 Hz, Ar-H), 7.04-6.96 (m, 2H, Ar-H), 5.44 (s, 1H, acetal-H), 4.25 (dd, 2H, *J* = 12, 4.8 Hz, -CH_2_-O-), 3.55 (t, 2H, *J* = 11 Hz, -CH_2_-O-), 2.21-2.10 (m, 1H, C_3_H_7_-CH-) 1.40–1.31 (m, 2H, CH_3_-CH_2_-), 1.13-1.08 (m, 2H, CH_2_H_5_-CH_2_-), 0.94 (t, 3H, *J* = 7.2 Hz, CH_3_-). ^13^C{^1^H} NMR (100 MHz, CDCl_3_, ppm): δ 161.06 (dd, *J* = 257, 6.7 Hz), 159.45 (d, *J* = 249 Hz), 158.94, 151.13 (ddd, *J* = 250, 11, 4.8 Hz), 144.86 (td, *J* = 12, 4.8 Hz), 142.33 (t, *J* = 13 Hz), 142.30 (d, *J* = 8.6 Hz), 138.4 (d, 249 Hz), 129.97 (d, *J* =1.9 Hz), 125.68 (d, *J* = 13 Hz), 122.59 (d, *J* = 2.9 Hz), 114.61 (d, *J* = 24 Hz), 112.92 (dd, *J* = 26, 3.4 Hz), 108.00 (t, *J* = 16 Hz), 107.16 (dd, *J* = 18, 6.3 Hz), 99.81, 72.64, 33.97, 30.32, 19.56, 14.21. ^19^F NMR (376 MHz, CDCl_3_, ppm): δ −109.6 (2F, 2,6-difluorobenzoate), −117.8 (1F, 3-fluorophenyl), −133.6 (2F, 3,4,5-trifluorpphenyl), −163.9 (1F, 3,4,5-trifluorophenyl). HRMS (FAB+) m/z: calcd for C_26_H_20_F_6_O_4_: 510.1266; found: 511.1343 (M+H), Error: −0.2 ppm. Anal. Calcd for C_26_H_20_F_6_O_4_: C 61.18, H 3.95%; found: C 61.06, H 3.96%.

4-4. Synthesis of 4-cyano-3,5-difluorophenyl 2,6-difluor-4-{2-fluoro-4-(*trans*-5-n-propyl-1,3-doxoane-2-yl)phenyl}benzoate (**DIO-bpp-4**)

To DCM (46 mL) were added compound **6** (5.36 g, 14.1 mmol), 2,6-difluoro-4-hydroxybenzonitrile (1.97 g, 13.3 mmol), DMAP (14.8 mg, 0.121 mmol). To the solution, WSC (2.55 g, 13.3 mmol) was added and then, the mixture was stirred at rt. for 2 h. After that, WSC (1.08 g, 5.63 mmol) was further added and then, the mixture was stirred at rt. overnight. The mixture was added in DCM and then washed with distilled water. The organic layer was dried over anhydrous MgSO_4_ and then concentrated. The residue was purified by silica-gel column chromatography (hexane/DCM volume ratio of 1/1) to afford **DIO-bpp-4** as a white slid in 2.77 g yield: (40.3%). ^1^H NMR (400 MHz, CDCl_3_, ppm): δ 7.47–7.36 (m, 3H, Ar-H), 7.26 (d, 2H, *J* = 10 Hz), 7.11 (dd, 2H, *J* = 10, 2.8 Hz, Ar-H), 5.45 (s, 1H, acetal-H), 4.26 (dd, 2H, *J* = 12, 4.6 Hz, -CH_2_-O), 3.56 (t, 2H, *J* = 11 Hz, -CH_2_-O), 2.21–2.10 (m, 1H, C_3_H_7_-CH-), 1.40–1.31 (m, 2H, CH_3_-CH_2_-), 1.14–1.08 (m, 2H, C_2_H_5_-CH_2_-), 0.94 (t, 3H, CH_3_-). ^13^C{^1^H} NMR (100 MHz, CDCl_3_, ppm): δ 163.57 (dd, *J* = 260, 5.8 Hz), 161.22 (dd, *J* = 258, 5.3 Hz), 159.45 (d, *J* = 258 Hz), 157.91, 155.14 (t, *J* = 13 Hz), 143.02 (t, *J* = 11 Hz), 142.50 (d, *J* = 7.7 Hz), 129.95, 125.50 (d, *J* = 13 Hz), 122.63, 114.66 (d, *J* = 24 Hz), 113.06 (dd, *J* = 23, 3.4 Hz), 108.63, 107.24, 106.90 (dd, *J* = 23, 3.9 Hz), 99.77, 90.57, 72.64, 33.98, 30.31, 19.56, 14.22. ^19^F NMR (376 MHz, CDCl_3_, ppm): δ −102.8 (2F, 4-cyano-3,5-difluorophenyl), −108.9 (2F, 2,6-difluorobenzoate), −117.8 (1F, 3-fluorophenyl). HRMS (FAB+) m/z: calcd for C_27_H_20_F_5_NO_4_: 517.1312; found: 518.1392 (M+H), Error: +0.2 ppm. Anal. Calcd for C_27_H_20_F_5_NO_4_: C 62.67, H 3.90, N 2.71%; found: C 62.80, H 3.92, N 2.64%.

4-5. Synthesis of 3,4,5-trifluorophenyl 4-(4-butyryloxy-2,6-difluorphenyl)-2-fluorobenzoate (**EST-bpp-1**)

4-5-1. 4-(2,6-difluoro-4-hydroxyphenyl)-2-fluorophenylbenzoic acid methyl ester (**7**): To a mixture of 1,4-dioxane (180 mL) and distilled water (10 mL) were added 4-bromo-3,5-difluorophenol (4.18 g, 20.0 mmol), 3-fluoro-4-methoxycarbonylphenylboronic acid (4.36 g, 22.0 mmol) and Pd(dppf)Cl_2_ DCM complex (490 mg, 0.600 mmol). After Cs_2_CO_3_ (19.5 g, 59.8 mmol) was added and then the mixture was stirred at 90 °C for 2 h in N_2_ atmosphere. After evaporation, the residue was dissolved in distilled water (300 mL) and then the pH of the solution was adjusted to ca. 7 by an addition of 1 M HCl aq. solution. The resulting precipitates were collected by suction filtration and washed with distilled water. The solid was dissolved in EtOAc and then insoluble parts were removed by suction filtration. After the filtrate was concentrated, the resulting solid was washed with DCM to afford a white solid in 2.78 g yield (49.3%). ^1^H NMR (400 MHz, DMSO-d_6_, ppm): δ 10.7 (br, 1H, OH), 7.69 (t, 1H, *J* = 8.0 Hz, Ar-H), 7.40–7.37 (m, 2H, Ar-H), 6.65-6.59 (m, 2H, Ar-H), 3.88 (s, 3H, CH_3_).

4-5-2. 4-(2,6-difluoro-4-hydroxyphenyl)-2-fluorophenylbenzoic acid (**8**): To a MeOH solution (100 mL) of compound **7** (2.78 g, 9.85 mmol) were added 5 M NaOH aq. (4 mL, 20 mmol) and distilled water (10 mL) and then, the solution was stirred at ambient temperature overnight. After evaporation of MeOH, the residue was added in distilled water (ca. 300 mL) and then the pH was adjusted to 2-3 by an addition of HCl solution. The solid was collected by suction filtration and washed with distilled water. After drying *in vacuo*, compound **8** was obtained as a white solid in 2.72 g yield (>99%). ^1^H NMR (400 MHz, DMSO-d_6_, ppm): δ 10.7 (br, 1H, OH), 7.94 (t, 1H, *J* = 8.0 Hz, Ar-H), 7.39-7.33 (m, 2H, Ar-H), 6.63 (d, 2H, *J* = 10 Hz, Ar-H). ^13^C{^1^H} NMR (100 MHz, DMSO-d_6_, ppm): δ 165.0 (d, *J* = 2.9 Hz), 161.0 (d, *J* = 256 Hz), 159.9 (t, *J* = 15 Hz), 160.1 (dd, *J* = 244, 11 Hz), 135.9 (d, *J* = 9.6 Hz), 132.0, 126.5, 118.7 (d, *J* = 14 Hz), 118.6, 106.7 (t, *J* = 18 Hz), 100.0–99.8 (m). ^19^F NMR (376 MHz, DMSO-d_6_, ppm): δ −111.0 (1F, 2-fluorobenzoic acid), −114.9 (2F, 2,6-difluorpphenyl).

4-5-3. 4-(4-butyryloxy-2,6-difluorophenyl)-2-fluorophenylbenzoic acid (**9**): Compound **8** (2.67 g, 9.85 mmol) was added in dehydrated pyridine (5 mL) and the mixture was heated at 60 °C. To the mixture, n-butyryl chloride (1.08 g, 10.1 mmol) was added dropwise and then the mixture was stirred at 60 °C for 1 h. After cooled to ambient temperature, the mixture was added into distilled water (300 mL) and then the pH of the aqueous solution was adjusted to ca. 3. The resulting precipitates were collected by suction filtration and then dissolved in DCM. The solution was dried over anhydrous MgSO_4_ and then concentrated to give compound **9** as a white solid in 2.32 g yield (69.7%). ^1^H NMR (400 MHz, DMSO-d_6_, ppm): δ 7.98 (t, 1H, *J* = 8.0 Hz, Ar-H), 7.52 (d, 1H, *J* = 11 Hz, Ar-H), 7.43 (d, 1H, *J* = 8.0 Hz, Ar-H), 7.26–7.21 (m, 2H, Ar-H), 2.60 (t, 2H, *J* = 7.2 Hz, -CH_2_-C=O-), 1.72–1.63 (m, 2H, CH_3_-CH_2_-), 0.98 (t, 3H, *J* = 7.2 Hz, CH_3_). ^13^C{^1^H} NMR (100 MHz, DMSO-d_6_, ppm): δ 168.6, 162.2 (d, *J* = 2.8 Hz), 158.2 (d, *J* = 256 Hz), 156.6 (dd, *J* = 247, 9.1 Hz), 149.0 (t, *J* = 12 Hz), 131.8 (d, *J* = 9.6 Hz), 129.5, 123.9, 116.8 (d, *J* = 11 Hz), 116.3 (d, *J* = 24 Hz), 110.9, 104.8–104.5 (m), 32.7, 15.2, 10.9. ^19^F NMR (376 MHz, DMSO-d_6_, ppm): δ −110.7 (1F, 2-fluorobenzoate), −113.6 (2F, 2,6-difluorophenyl).

4-5-4. **EST-bpp-1**: To DCM (20 mL) were added compound **9** (1.01 g, 3.00 mmol), 3,4,5-trifluorophenol (503 mg, 3.40 mmol) and DMAP (37 mg, 0.30 mmol). To the mixture, WSC (633 mg, 3.30 mmol) was added and then, the mixture was stirred at ambient temperature for 15 min. After that, the mixture was added in DCM (100 mL) and then washed with distilled water (100 mL × 2), followed by ultra-pure water (100 mL ×2). After drying over anhydrous Na_2_SO_4_, the organic layer was concentrated and washed with MeOH. The solid was dissolved in DCM and the insoluble parts were removed by centrifugation (10,000 rpm, 15 °C, 10 min). The crude product was purified by silica-gel column chromatography (hexane/DCM volume ratio of 1/1), followed by recrystallization from MeOH/acetone to give **EST-bpp-1** as a white solid in 511 mg yield (36.4%). ^1^H NMR (400 MHz, CDCl_3_, ppm): δ 8.14 (t, 1H, *J* = 7.6 Hz, Ar-H), 7.40 (dd, 1H, *J* = 8.4, 1.4 Hz, Ar-H), 7.36 (dd, 1H, *J* = 11, 1.4 Hz, Ar-H), 7.02–6.94 (m, 2H, Ar-H), 6.90–6.85 (m, 2H, Ar-H), 2.58 (t, 3H, *J* = 7.2 Hz, -CH_2_-C=O-), 1.83–1.76 (m, 2H, CH_3_-CH_2_-), 1.06 (t, 3H, *J* = 7.2 Hz, CH_3_-). ^13^C{^1^H} NMR (100 MHz, CDCl_3_, ppm): δ 171.20, 161.97 (d, *J* = 261 Hz), 161.64 (d, *J* = 3.9 Hz), 159.74 (dd, *J* = 250, 8.6 Hz), 151.74 (t, *J* = 14 Hz), 151.14 (ddd, *J* = 250, 11, 5.3 Hz), 145.16 (td, *J* = 11, 4.5 Hz), 138.32 (dt, *J* = 249, 15 Hz), 136.70 (d, *J* = 9.6 Hz), 132.36, 126.34, 119.32 (d, *J* = 23 Hz), 116.62 (d, *J* = 9.6 Hz), 113.60 (t, *J* = 19 Hz), 107.19 (dd, *J* = 18, 6.2 Hz), 106.53 (dd, *J* = 22, 8.2 Hz), 36.09, 18.30, 13.59. ^19^F NMR (376 MHz, CDCl_3_, ppm): δ −108.6 (1F, 2-fluorobenzoate), −113.5 (2F, 3,5-difluorophenolate), −133.8 (2F, 3,4,5-trifluorpphenyl), −164.2 (1F, 3,4,5-trifluorophenyl). HRMS (FAB+) m/z: calcd for C_23_H_14_F_6_O_4_: 468.0796; found: 469.0877 (M+H), Error: +0.5 ppm. Anal. Calcd for C_23_H_14_F_6_O_4_: C 58.98, H 3.01%; found: C 59.05, H 3.02%.

4-6. Synthesis of 3,5-diifluoro-4-cyanophenyl 4-(4-butyryloxy-2,6-difluorphenyl)-2-fluorobenzoate (**EST-bpp-2**)

To a DCM (10 mL) containing compound **9** (1.18 g, 3.50 mmol) was added WSC (814 mg, 4.25 mmol) and then, the mixture was stirred for 15 min, resulting in a clear solution. To the solution, 4-hydroxy-2,6-difluorobenzonitrile (597 mg, 3.85 mmol) was added and then, the mixture was stirred overnight. After an addition of DCM, the solution was washed with distilled water (250 mL × 2), followed by ultra-pure water (200 mL) and then, the organic layer was dried over anhydrous Na_2_SO_4_. After evaporation, the residue was purified by silica-gel column chromatography (hexane/DCM volume ratio of 1/3 to 1/5) followed by recrystallization from MeOH. The obtained solid was further purified by the recrystallization from hexane/EtOAc to give **EST-bpp-2** as a white solid in 709 mg yield (42.7%). ^1^H NMR (400 MHz, CDCl_3_, ppm): δ 8.15 (t, 1H, *J* = 7.6 Hz, Ar-H), 7.43 (dd, 1H, *J* = 8.0, 1.2 Hz, Ar-H), 7.38 (dd, 1H, *J* = 11, 1.6 Hz, Ar-H), 7.12-7.09 (m, 2H, Ar-H), 6.91-6.86 (m, 2H, Ar-H), 2.59 (t, 3H, *J* = 7.4 Hz, -CH_2_-C=O-), 1.85-1.76 (m, 2H, CH_3_-CH_2_-), 1.06 (t, 3H, *J* = 7.4 Hz, CH_3_-). ^13^C{^1^H} NMR (100 MHz, CDCl_3_, ppm): δ 171.19, 163.57 (dd, *J* = 261, 6.7 Hz), 162.06 (d, *J* = 262 Hz), 159.72 (dd, *J* = 249, 8.7 Hz), 160.64 (d, *J* = 3.9 Hz), 155.54 (t, *J* = 13 Hz), 151.87 (t, *J* = 14 Hz), 137.31 (d, *J* = 9.6 Hz), 132.43, 126.50, 119.44 (d, *J* = 24 Hz), 115.94 (d, *J* = 9.5 Hz), 113.43 (t, *J* = 18 Hz), 108.71, 106.96 (dd, *J* = 23, 3.9 Hz), 106.59 (dd, *J* = 22, 8.2 Hz), 90.34 (t, *J* = 20 Hz), 36.08, 18.29, 13.59. ^19^F NMR (376 MHz, CDCl_3_, ppm): δ −103.0 (2F, 4-cyano-3,5-difluorophenyl), −108.0 (1F, 2-fluorobenzoate), −113.5 (2F, 3,5-difluorophenolate). HRMS (FAB+) m/z: calcd for C_24_H_14_F_5_NO_4_: 475.0843; found: 476.0923 (M+H), Error: +0.4 ppm. Anal. Calcd for C_24_H_14_F_5_NO_4_: C 60.64, H 2.97, N 2.95%; found: C 60.77, H 2.97, N 2.93%.

4-7. Synthesis of 3,4,5-trifluorophenyl 4-(4-butyryloxy-2-fluorphenyl)-2,6-difluorobenzoate (**EST-bpp-3**)

4-7-1. benzyl 4-bromo-3-fluorophenyl ether (**10**): To THF (15 mL) of 4-bromo-3-fluorophenol (2.87 g, 15.0 mmol), benzyl bromide (2.82 g, 16.5 mmol) was added K_2_CO_3_ (4.15 g, 30.0 mmol) and then, the mixture was stirred at 60°C overnight. The solids were removed by suction filtration and then, the filtrate was dried over anhydrous MgSO_4_. After evaporation, compound **10** was obtained as colorless liquid in 4.49 g yield (>99%). ^1^H NMR (400 MHz, CDCl_3_, ppm): δ 7.41–7.33 (m, 6H, Ar-H), 6.76 (dd, 1H, *J* = 10, 2.8 Hz, Ar-H), 6.67 (ddd, 1H, *J* = 9.0, 2.8, 1.4 Hz, Ar-H), 5.02 (s, 2H, CH_2_). ^13^C{^1^H} NMR (100 MHz, CDCl_3_, ppm): δ 159.4 (d, *J* = 245 Hz), 159.3 (d, *J* = 9.6 Hz), 136.0, 133.4 (d, *J* = 1.9 Hz), 128.7, 128.3, 127.4, 112.2 (d, *J* = 2.9 Hz), 103.8 (d, *J* = 25 Hz), 99.6 (d, *J* = 21 Hz). ^19^F NMR (376 MHz, CDCl_3_, ppm): δ −106.4 (1F, Ar-F).

4-7-2. 4-(4-benzyloxy-2-fluorophenyl)-2,6-difluorobenzene (**11**): To DMF (56 mL) were added compound **10** (4.49 g, 16.0 mmol), 3,5-difluorophenylboronic acid (3.28 g, 20.7 mmol), [Pd(PPh_3_)_4_] (0.369 g, 0.319 mmol, 2 mol%), K_3_PO_4_ (6.77g, 31.9 mmol) and distilled water (16 mL). The mixture was stirred under Ar atmosphere at 100 °C overnight and then added in distilled water. The resulting precipitates were collected by suction filtration and then, dried under reduced pressure to give a white solid in 4.88 g yield (97.0%). ^1^H NMR (400 MHz, CDCl_3_, ppm): δ 7.45–7.31 (m, 6H, Ar-H), 7.06–7.03 (m, 2H, Ar-H), 6.86–6.75 (m, 3H, Ar-H), 5.10 (s, 2H, CH_2_).

4-7-3. 4-(4-benzyloxy-2-fluorophenyl)-2,6-difluorobenzoic acid (**12**): Compound **11** (4.72 g, 15.0 mmol) was dissolved in dehydrated THF (34.1 mL) and the solution was cooled to −78 °C under Ar condition. To the solution, 1.6 M n-BuLi n-hexane solution (11.6 mL, 18.6 mmol) was added dropwise and then, the solution was stirred at −78 °C for several minutes. At the temperature, dry ice blocks (12.8 g, 291 mmol) was added and then, the mixture was stirred for 15 min. After that, the mixture was annealed to rt. and then, stirred for 2 h. After quenching by distilled water and HCl aq. solution, the mixture was concentrated and then, the resulting solids were collected by suction filtration and then washed with distilled water. After dying, a gray solid was obtained in 5.15 g yield (95.9%). ^1^H NMR (400 MHz, CDCl_3_, ppm): δ 7.45–7.34 (m, 6H, Ar-H), 7.18 (d, 2H, *J* = 11 Hz, Ar-H), 6.88 (dd, 1H, *J* = 8.8, 2.8 Hz, Ar-H), 6.81 (dd, 1H, *J* = 13, 2.0 Hz, Ar-H), 5.11 (s, 2H, CH_2_).

4-7-4. 3,4,5-trifluorophenyl 4-(4-benzyloxy-2-fluorophenyl)-2,6-difluorobenzoate (**13**): To DCM (55 mL) were added compound **12** (5.15 g, 14.4 mmol), 3,4,5-trifluorophenol (2.35 g, 15.8 mmol), DMAP (17.6 mg, 0.144 mmol) and WSC (3.04 g, 15.9 mmol). After the mixture was stirred for 2 h, WSC (1.29 g, 6.74 mmol) was added and then the mixture was further stirred overnight. The mixture was added in DCM and then washed with distilled water. After dried over anhydrous MgSO_4_, the organic layer was concentrated. The crude product was purified by silica-gel column chromatography (hexane/DCM volume ratio of 1/1) to give a white solid in 3.25 g yield (46.2%). ^1^H NMR (400 MHz, DMSO-d_6_, ppm): δ 7.60 (t, 1H, *J* = 9.2 Hz, Ar-H), 7.48–7.33 (m, 9H, Ar-H), 7.10 (dd, 1H, *J* = 13, 2.2 Hz, Ar-H), 7.00 (dd, 1H, *J* = 8.6 2.2 Hz, Ar-H), 5.76 (s, 2H, CH_2_).

4-7-5. 3,4,5-trifluorophenyl 2,6-difluoro-4-(2-fluoro-4-hydroxyphenyl)benzoate (**14**): To a THF solution of compound **13** (3.25 g, 6.64 mmol) was added Pd/C (162 mg) and then, the mixture was stirred under H_2_ atmosphere at rt. overnight. After removal of Pd/C by suction filtration, the filtrates were concentrated and then, the reaction was repeated once at the same condition. The crude product was purified by silica-gel column chromatography (DCM) followed by recrystallization to afford a white solid in 1.06 g yield (40.1%). ^1^H NMR (400 MHz, DMSO-d_6_, ppm): δ 10.5 (br, 1H, OH), 7.59–7.50 (m, 5H, Ar-H), 6.78–6.72 (m, 2H, Ar-H).

4-7-6. **EST-bpp-3**: To a THF solution (26 mL) of compound **14** (1.02 g, 2.56 mmol) and NEt_3_ (0.29 g, 2.9 mmol) was added butyryl chloride (0.29 g, 2.7 mmol) and then, the mixture was stirred for 1 h. After removal of precipitates by suction filtration, the filtrates were concentrated and then, the crude product was purified by silica-gel column chromatography (hexane/DCM volume ratio of 1:1). After drying, a white solid was obtained in 1.05 g yield (87.6%). ^1^H NMR (400 MHz, CDCl_3_, ppm): δ 7.48-7.44 (m, 1H, Ar-H), 7.23 (dd, 2H, *J* = 10, 0.8 Hz, Ar-H), 7.06-6.97 (m, 4H, Ar-H), 2.58 (t, 2H, *J* = 7.4 Hz, -CH_2_-C=O-), 1.85-1.76 (m, 2H, CH_3_-CH_2_-), 1.06 (t, 3H, *J* = 7.4 Hz, CH_3_-). ^13^C{^1^H} NMR (100 MHz, CDCl_3_, ppm): δ 171.53, 161.11 (dd, *J* = 257, 6.2 Hz), 159.55 (d, *J* = 251 Hz), 158.91, 152.39 (d, *J* = 12 Hz), 151.16 (ddd, *J* = 251, 11, 4.8 Hz), 144.85 (td, *J* = 12, 3.6 Hz), 141.86 (t, *J* = 12 Hz), 138.47 (dt, *J* = 250, 15 Hz), 130.49 (d, *J* = 3.6 Hz), 123.06 (d, *J* = 13 Hz), 118.40 (d, *J* = 3.8 Hz), 112.86 (dd, *J* = 24, 6.4 Hz), 110.82 (d, *J* = 26 Hz), 108.08 (t, *J* = 16 Hz), 107.16 (dd, *J* = 18, 6.3 Hz), 36.15, 18.36, 13.62. ^19^F NMR (376 MHz, CDCl_3_, ppm): δ −109.4 (2F, 2,6-difluorobenzoate), −115.0 (1F, 3-fluorophenolate), −133.6 (2F, 3,4,5-trifluorpphenyl), −163.8 (1F, 3,4,5-trifluorophenyl). HRMS (FAB+) m/z: calcd for C_23_H_14_F_6_O_4_: 468.0796; found: 469.0878 (M+H), Error: +0.7 ppm. Anal. Calcd for C_23_H_14_F_6_O_4_: C 58.98, H 3.01%; found: C 59.12, H 3.04%.

4-8. Synthesis of 4-cyano-3,5-difluorophenyl 4-(4-butylyloxy-2-fluorophenyl)-2,6-difluorobenzoate (**EST-bpp-4**)

4-8-1. 2,6-difluoro-4-(2-fluoro-4-hydroxyphenyl)benzoic acid (**15**): To a THF solution (122 mL) of compound **12** (4.03 g, 11.3 mmol) was added Pd/C (202 mg) and then, the solution was stirred at rt. for 1 d under H_2_ atmosphere. After the removal of Pd/C by suction filtration, the filtrate was concentrated. After drying, crude product obtained was purified by silica-gel column chromatography (DCM) followed by recrystallization to afford a white solid in 1.62 g yield (53.6%).

4-8-2. 4-(4-butylyloxyphenyl-2-fluoro)-2,6-difluorobenzoic acid (**16**): To a THF solution (46 mL) of compound **15** (1.24 g, 4.62 mmol) and NEt_3_ (707 mg, 6.99 mmol) was added butyryl chloride (499 mg, 4.68 mmol). After the solution was stirred at rt. for 1 h, solids were removed by suction filtration. The filtrate was concentrated and then, the resulting solid was purified by silica-gel column chromatography (EtOAc/MeOH volume ratio of 4:1). After drying, a white solid was obtained in 1.32 g yield (84.3%).

4-8-3. **EST-bpp-4:** To a DCM solution (15 mL) of compound **16** (1.32 g, 3.90 mmol) and 2,6-difluoro-4-hydroxybenzonitrile (0.667 g, 4.30 mmol) was added WSC (1.20 g, 6.26 mmol) and then, the mixture was stirred at rt. for 2 h. The mixture was added in DCM and then, washed with distilled water. After drying over anhydrous MgSO_4_, the organic layer was concentrated. The residue was purified by silica-gel column chromatography (hexane/DCM volume ratio of 1:1) followed by recrystallization to give a white solid. ^1^H NMR (400 MHz, CDCl_3_, ppm): δ 7.49-7.45 (m, 1H, Ar-H), 7.26 (d, 2H, *J* = 10 Hz, Ar-H), 7.12 (dd, 2H, *J* = 10, 2.8 Hz, Ar-H), 7.07–7.03 (m, 2H, Ar-H), 2.59 (t, 2H, *J* = 7.4 Hz, -CH_2_-C=O-), 1.86–1.76 (m, 2H, CH_3_-CH_2_-), 1.07 (t, 3H, *J* = 7.2 Hz, CH_3_-). ^13^C{^1^H} NMR (100 MHz, CDCl_3_, ppm): δ 171.52, 163.57 (dd, *J* = 260, 6.3 Hz), 161.27 (dd, *J* = 259, 5.7 Hz), 159.55 (d, *J* = 251 Hz), 157.89, 155.13 (t, *J* = 13 Hz), 152.52 (d, *J* = 12 Hz), 142.52 (t, *J* = 11 Hz), 130.47 (t, *J* = 3.8 Hz), 122.87 (d, *J* = 13 Hz), 118.47 (d, *J* = 2.9 Hz), 112.99 (dd, *J* = 23, 6.7 Hz), 110.87 (d, *J* = 26 Hz), 108.61, 107.32 (t, *J* = 16 Hz), 106.90 (dd, *J* = 23, 3.9 Hz), 90.61 (t, *J* = 19 Hz), 36.14, 18.35, 13.62. ^19^F NMR (376 MHz, CDCl_3_, ppm): δ −102.7 (2F, 4-cyano-3,5-difluorophenyl), −108.7 (2F, 2,6-difluorobenzoate), −114.9 (1F, 3-fluorophenolate). HRMS (FAB+) m/z: calcd for C_24_H_14_F_5_NO_4_: 475.0843; found: 476.0925 (M+H), Error: +0.8 ppm. Anal. Calcd for C_24_H_14_F_5_NO_4_: C 60.64, H 2.97, N 2.95%; found: C 60.74, H 2.98, N 2.95%.

4-9. Synthesis of 3,5-difluoro-4-(3,4,5-trifluorophenyl)phenyl 4-butylyloxy-2-fluorobenzoate (**EST-1’**)

4-9-1. Allyl 4-bromo-2,6-difluorophenyl ether (**17**): To an acetone solution (20 mL) of 4-bromo-3,5-difluorophenol (4.81 g, 23.0 mmol) was added K_2_CO_3_ (4.77 g, 34.5 mmol) an allyl bromide (3.00 g, 24.8 mmol). After refluxed for 3 h, the mixture was filtrated and then, the filtrate was concentrated. The resulting liquid was dissolved in EtOAc and then washed three times with distilled water. After drying over anhydrous Na_2_SO_4_, pale yellow liquids were obtained. Yield: 5.45 g (95.1%). ^1^H NMR (400 MHz, CDCl_3_, ppm): δ 6.57–6.53 (m, 2H, Ar-H), 6.02-5.96 (1H, m, H_2_C=CH-), 5.43–5.31 (m, 2H, H_2_C=CH-), 4.51–4.49 (m, 2H, -CH_2_-O-).

4-9-2. 3,5-Difluoro-4-(3,4,5-trifluorophenyl)phenol (**18**): To DMF (3.5 mL) were added compound **17** (2.49 g, 10.0 mmol), 3,4,5-trifluorophenylbronic acid (2.29 g, 13.0 mmol), Pd(PPh_3_)_4_ 120 mg (0.104 mmol), and K_3_PO_4_ (4.24 g, 20.0 mmol). After stirred at 100 °C in a N_2_ condition overnight, the mixture was added in H_2_O (450 mL). The resulting precipitates were collected by suction filtration and then recrystallized from DCM to afford pale yellow crystals. Yield: 1.16 g (44.6%). ^1^H NMR (400 MHz, DMSO-d_6_, ppm): δ 10.7 (br, 1H, OH), 7.43–7.40 (t, 2H, *J* = 7.6 Hz, Ar-H), 6.62–6.57 (m, 2H, Ar-H).

4-9-3. Methyl 4-benzyloxy-3-fluorobenzoate (**19**): To THF (40 mL) were added methyl 2-fluoro-4-hydroxybenzoate (4.30 g, 25.3 mmol), benzyl bromide (4.32 g, 25.3 mmol), and K_2_CO_3_ (4.84 g, 35.0 mmol). The mixture was stirred at 60 °C for 1 d and then filtrated to remove solids. The filtrate was dried and then the resulting solids were purified by silica-gel column chromatography (hexane/DCM volume ratio of 1:1 to 1:4). After washing with hexane, a white solid was obtained. Yield: 5.30 g (80.6%). ^1^H NMR (400 MHz, DMSO-d_6_, ppm): δ 7.91 (t, 1H, *J* = 8.6 Hz, Ar-H), 7.43-7.33 (m, 5H, Ar-H), 6.79 (dd, 1H, *J* = 8.8, 2.4 Hz, Ar-H), 6.71 (dd, 1H, *J* = 13, 2.4 Hz, Ar-H), 5.10 (s, 2H, CH_2_), 3.90 (s, 3H, CH_3_).

4-9-4. 4-Benzyloxy-3-fluorobenzoic acid (**20**): Compound **19** (5.29 g, 20.3 mmol) was dissolved in a mixed solution of MeOH (20 mL) and THF (20 mL). To the solution, 5 M NaOH aq. (6.0 mL, 30 mmol) and H_2_O (20 mL) were added and then, the mixture was stirred at rt. for 3 h. After evaporation of organic solvents, the aqueous solution was diluted by H_2_O and then acidified (PH = ca. 3) by an addition of 1 M HCl aq. The resulting precipitates were collected by suction filtration and washed with distilled water. After drying under reduced pressure, a white solid was obtained. Yield: 4.54 g (90.6%). ^1^H NMR (400 MHz, DMSO-d_6_, ppm): δ 7.91 (t, 1H, *J* = 8.6 Hz, Ar-H), 7.47–7.34 (m, 5H, Ar-H), 7.00 (dd, 1H, *J* = 13, 2.4 Hz, Ar-H), 6.94 (dd, 1H, *J* = 8.8, 2.4 Hz, Ar-H), 5.20 (s, 2H, CH_2_).

4-9-5. 2,3-Difluoro-4-(3,4,5-trifluorophenyl)phenyl 4-benzyloxy-3-fluorobenzoate (**21**): To a DCM solution of compounds **18** (531 mg, 2.04 mmol) and **20** (503 mg, 2.04 mmol) were added DMAP (244 mg, 2.00 mmol) and WSC (502 mg, 2.62 mmol). The mixture was stirred at rt. for 2 d and then the precipitates (compound **21**) were collected by suction filtration. The filtrate (100 mL) was washed with diluted HCl aq. (100 mL), followed by distilled water (100 mL × 2). After drying over anhydrous Na_2_SO_4_ followed by evaporation, the resulting solids were washed by MeOH. After drying, a white solid was obtained. Yield: 763 mg (76.6%). ^1^H NMR (400 MHz, CDCl_3_, ppm): δ 8.04 (t, 1H, *J* = 8.6 Hz, Ar-H), 7.43–7.35 (m, 5H, Ar-H), 7.12 (t, 1H, *J* = 7.2 Hz, Ar-H), 6.99–6.94 (m, 2H, Ar-H), 6.88 (dd, 1H, *J* = 8.4, 2.4 Hz, Ar-H), 6.79 (dd, 1H, *J* = 13, 2.6 Hz, Ar-H), 5.15 (s, 2H, CH_2_). ^13^C{^1^H} NMR (100 MHz, CDCl_3_, ppm): δ 164 (d, J = 262 Hz), 164.8 (d, *J* = 12 Hz), 161.5 (d, *J* = 3.8 Hz), 159.7 (dd, *J* = 249, 8.6 Hz), 151.2 (d, *J* = 14 Hz), 151.0 (ddd, *J* = 247, 9.6, 4.3 Hz), 139.7 (dt, *J* = 253, 14 Hz), 135.3, 134.0, 128.8, 127.5, 124.3 (td, *J* = 9.1, 3.8 Hz), 114.9–114.7 (m), 113.4, 111.4, 109.3 (d, *J* = 8.6 Hz), 106.8–106.5 (m), 103.5 (d, *J* = 26 Hz), 70.7. ^19^F NMR (376 MHz, CDCl_3_, ppm): δ −103.5 (1F, 2-fluorobenzoate), −112.6 (2F, 3,5-difluorophenolate), −134.3 (2F, 3,4,5-trifluorophenyl), −160.0 (1F, 3,4,5-trifluorophenyl).

4-9-6. 2,3-Difluoro-4-(3,4,5-trifluorophenyl)phenyl 3-fluoro-4-hydroxybenzoate (**22**): To THF (15 mL) were added compound **21** (665 mg, 1.36 mmol) and 5wt% Pd/C (80 mg). The mixture was stirred at rt. in H_2_ condition for 14 h. After removal of solids by celite filtration, the filtrate was concentrated. The resulting solids were purified by reprecipitation from hexane/acetone to afford a white solid. Yield: 483 mg (89.1%). ^1^H NMR (400 MHz, acetone-d_6_, ppm): δ 10.0 (br, 1H, OH), 8.02 (t, 1H, *J* = 8.6 Hz, Ar-H), 7.45 (t, 1H, *J* = 7.6 Hz, Ar-H), 7.24–7.19 (m, 2H, Ar-H), 6.86 (dd, 1H, *J* = 8.8, 2.4 Hz, Ar-H), 6.77 (dd, 1H, *J* = 13, 2.4 Hz, Ar-H). ^13^C{^1^H} NMR (100 MHz, acetone-d_6_, ppm): δ 164.6 (d, *J* = 12 Hz), 164.2 (d, *J* = 259 Hz), 161.1 (d, *J* = 5.7 Hz), 159.8 (dd, *J* = 247, 9.1 Hz), 152.1 (t, *J* = 15 Hz), 150.9 (ddd, *J* = 247, 10, 4.6 Hz), 134.3, 125.2, 115.3–115.1 (m), 113.1, 112.2, 108.1 (d, *J* = 9.6 Hz), 107.1–106.8 (m), 103.9 (d, *J* = 24 Hz).

4-9-7. **EST-1’**: To DCM (10 mL) of compound **22** (335 mg, 0.841 mmol), a DCM solution (1 mL) of NEt_3_ (125 mg, 1.24 mmol) and a DCM solution (1 mL) of butylyl chloride (121 mg, 1.14 mmol) were added slowly in an ice bath. The mixture was stirred at rt. for 15 h and then diluted by DCM (50 mL) and then washed with distilled water (50 mL) followed by ultra-pure water (50 mL ×2). After drying over anhydrous Na_2_SO_4_, the DCM layer was concentrated. The resulting solid was reprecipitated from acetone/MeOH and then purified by silica-gel column chromatography (hexane/DCM volume ratio of 1:1 to 1:2). The solid was further purified by recrystallization from hexane/EtOAc to give white crystals. Yield: 318 mg (80.7%). ^1^H NMR (400 MHz, CDCl_3_, ppm): δ 8.14–8.10 (m, 1H, Ar-H), 7.15–7.06 (m, 4H, Ar-H),7.01–6.96 (m, 2H, Ar-H), 2.59 (t, 2H, *J* = 7.4 Hz, -C=O-CH_2_-), 1.85–1.76 (m, 2H, CH_3_-CH_2_-), 1.06 (t, 3H, *J* = 7.2 Hz, CH_3_-). ^13^C{^1^H} NMR (100 MHz, CDCl_3_, ppm): δ 171.0, 162.9 (d, *J* = 263 Hz), 161.2 (d, *J* = 4.8 Hz), 159.7 (dd, *J* = 249, 8.7 Hz),156.2 (d, *J* = 12 Hz), 151.1 (ddd, *J* = 248, 11, 3.9 Hz), 151.0 (t, *J* = 14 Hz), 139.8 (dt, *J* = 253, 15 Hz), 133.5, 124.2 (td, *J* = 9.0, 4.8 Hz), 117.9 (d, *J* = 2.9 Hz), 114.9–114.7 (m), 114.3 (d, *J* = 9.6 Hz), 113.7 (t, *J* = 17 Hz), 106.8–106.5 (m), 36.1, 18.3, 13.6. ^19^F NMR (376 MHz, CDCl_3_, ppm): δ −105.0 (1F, 2-fluorobenzoate), −113.6 (2F, 3,5-difluorophenolate), −133.5 (2F, 3,4,5-trifluorophenyl), −161.2 (1F, 3,4,5-trifluorophenyl). HRMS (FAB+) m/z: calcd for C_23_H_14_F_6_O_4_: 468.0796; found: 468.0798 (M), Error: +0.4 ppm. Anal. Calcd for C_23_H_14_F_6_O_4_: C 58.98, H 3.01%; found: C 59.12, H 2.85%.

4-10. Synthesis of 3,4,5-trifluorophenyl 4-(4-butylyloxy-2-fluorophenyl)-2-fluorobenzoate (**EST-bpp-5**)

4-10-1. Methyl 4-(4-benzyloxy-3-fluorophenyl)-3-fluorobenzoate (**23**): To a mixed solution of 1,4-dioxane (90 mL) and H_2_O (10 mL) were added compound **10** (5.62 g, 20.0 mmol), 3-fluoro-4-methoxycarbonylphenylboronic acid (4.36 g, 22.0 mmol), Pd(dppf)Cl_2_ (439 mg, 0.600 mmol, 3 mol%), and Cs_2_CO_3_ (19.5 g, 59.8 mmol). The mixture was stirred at 90 °C for 2.5 h and then solvents were removed by evaporation. H_2_O (200 mL) were added to the resulting solids and then the mixture was neutralized by an addition of HCl aq. The solid was collected by suction filtration and then washed with MeOH. The crude product (5.58 g) was purified by silica-gel column chromatography (hexane/DCM volume ratio of 1:1 to 1:2), followed by washing with hexane to afford a white solid. Yield: 4.80 g (67.7%). ^1^H NMR (400 MHz, CDCl_3_, ppm): δ 7.97 (t, 1H, *J* = 8.0 Hz, Ar-H), 7.45–7.25 (m, 8H, Ar-H), 6.85 (dd, 1H, *J* = 8.8, 2.8 Hz, Ar-H), 6.79 (dd, 1H, *J* = 13, 2.6 Hz, Ar-H), 5.09 (s, 2H, -CH_2_-), 3.94 (s, 3H, CH_3_-). ^13^C{^1^H} NMR (100 MHz, CDCl_3_, ppm): δ 164.8 (d, *J* = 3.8 Hz), 161.9 (d, *J* = 258 Hz), 160.4 (d, *J* = 12 Hz), 160.4 (d, *J* = 248 Hz), 142.4 (d, *J* = 9.6 Hz), 136.1, 132.1, 130.8 (d, *J* = 4.8 Hz), 128.7, 128.3, 127.5, 124.1 (t, *J* = 3.4 Hz), 119.2 (d, *J* = 13 Hz), 117.1–116.8 (m), 111.5 (d, *J* = 2.9 Hz), 103.2 (d, *J* = 258 Hz), 70.4, 52.3. ^19^F NMR (376 MHz, CDCl_3_, ppm): δ −110.7 (1F, 2-fluorobenzoate), −115.6 (1F, 2-fluorophenolate).

4-10-2. 4-(4-benzyloxy-3-fluorophenyl)-3-fluorobenzoic acid (**24**): Compound **23** (4.61 g, 13.0 mmol) was added in a mixed solvent of THF (50 mL) and MeOH (50 mL). To the mixture, 5 M NaOH aq. (10 mL, 50 mmol) was added and then the mixture was stirred at 60 °C for 3 h. After evaporation, the solid was washed with H_2_O followed by THF. The solid was dispersed in H_2_O and then the mixture was acidified (pH < 3) by an addition of HCl aq. After filtration, the solid was dispersed in MeOH and the mixture was acidified by an addition of HCl aq. After filtration, the solid was dried under reduced pressure to afford a white solid. Yield: 4.53 g (>99%). --). The solid was used for the next synthetic step without any further purification due to its poor solubility. IR (ATR): ν =3300 (m; ν(O-H) of COOH), 3061–3035 (w; ν(C-H of aromatic)), 2909–2869 (w; ν(C-H) of methylene), 1589 (s; ν(C=O) of COOH), 1518–1436 (m; δ(C–C) of aromatic), 795–696 cm^−1^ (s; δ (C-H) of aromatic).

4-10-3. 3,4,5-trifluorophenyl 4-(4-benzyloxy-3-fluorophenyl)-3-fluorobenzoate (**25**): To DCM (10 mL) were added compound **24** (2.72 g, 8.00 mmol), 3,4,5-trifluorophenol (1.35 g, 8.80 mmol), DMAP (195 mg, 1.60 mmol). To the mixture, WSC (1.83 g, 9.55 mmol) was added and then the mixture was stirred at rt. for 3 d. The mixture was added in DCM (100 mL) and then washed with H_2_O (100 mL ×3). After drying over anhydrous Na_2_SO_4_, the DCM layer was concentrated and then the resulting solid was purified by reprecipitation from acetone/MeOH to give a white solid. Yield: 3.35 g (89.1%). ^1^H NMR (400 MHz, CDCl_3_, ppm): δ 8.08 (t, 1H, *J* = 7.8 Hz, Ar-H), 7.45–7.34 (m, 8H, Ar-H), 7.00–6.93 (m, 2H, Ar-H), 6.88 (dd, 1H, *J* = 8.4, 2.4 Hz, Ar-H), 6.81 (dd, 1H, *J* = 13, 2.4 Hz, Ar-H), 5.11 (s, 2H, -CH_2_-). ^13^C{^1^H} NMR (100 MHz, CDCl_3_, ppm): δ 162.3 (d, *J* = 261 Hz), 161.8 (d, *J* = 4.8 Hz), 160.7 (d, *J* = 12 Hz), 160.5 (d, *J* = 249 Hz), 151.1 (ddd, *J* = 249, 11, 5.5 Hz), 145.2 (td, *J* = 12, 4.5 Hz), 143.9 (d, *J* = 9.6 Hz), 143.2 (dt, *J* = 249, 16 Hz), 136.0, 132.5, 130.8 (d, *J* = 4.8 Hz), 128.8, 128.4, 127.5, 124.4 (t, *J* = 3.4 Hz), 118.8 (d, *J* = 12 Hz), 117.3 (dd, *J* = 23, 3.9 Hz), 115.1 (d, *J* = 9.6 Hz), 111.6 (d, *J* = 2.9 Hz), 107.3–107.1 (m), 103.3 (d, *J* = 27 Hz), 70.5.

4-10-4. 3,4,5-trifluorophenyl 4-(4-hydroxy-3-fluorophenyl)-3-fluorobenzoate (**26**): To a THF solution (30 mL) of compound **25** (3.30 g, 7.02 mmol) was added 5 wt% Pd/C (367 mg). The mixture was stirred at rt. overnight and then the solids was removed by celite filtration. The filtrate was concentrated and dried to give a white solid. Yield: 2.62 g (98.5%). ^1^H NMR (400 MHz, DMSO-d_6_, ppm): δ 10.4 (br, 1H, OH), 8.12 (t, 1H, *J* = 8.0 Hz, Ar-H), 7.57-7.49 (m, 5H, Ar-H), 6.79-6.71 (m, 2H, Ar-H). ^13^C{^1^H} NMR (100 MHz, DMSO-d_6_, ppm): δ 162.1 (d, *J* = 259 Hz), 161.6 (d, *J* = 3.9 Hz), 160.6 (d, *J* = 12 Hz), 160.5 (d, *J* = 246 Hz), 150.7 (ddd, *J* = 246, 11, 4.8 Hz), 146.0 (td, *J* = 12, 4.3 Hz), 144.1 (d, *J* = 8.6 Hz), 137.9 (dt, *J* = 246, 14 Hz), 133.1, 131.9 (d, *J* = 4.8 Hz), 125.0, 117.0 (dd, *J* = 23, 3.8 Hz), 166.6 (d, *J* = 12 Hz), 115.1 (d, *J* = 8.6 Hz), 113.1, 108.9-108.7 (m), 103.8 (d, *J* = 23 Hz). ^19^F NMR (376 MHz, DMSO-d_6_, ppm): δ −109.0 (1F, 2-fluorobenzoate), −115.7 (1F, 2-fluorophenolate), −134.4 (2F, 3,4,5-trifluorophenyl), −164.5 (1F, 3,4,5-trifluorophenyl).

4-10-5. **EST-bpp-5**: To DCM (30 mL) were added compound **26** (761 mg, 2.00 mmol) and NEt_3_ (220 mg, 2.17 mmol). To the mixture, a DCM solution (2 mL) of butylyl chloride (213 mg, 2.00 mmol) was added at 0 °C and then the mixture was stirred at rt. overnight. The mixture was added in DCM (50 mL) and then washed with H_2_O (50 mL ×3). After drying over anhydrous Na_2_SO_4_, the DCM layer was concentrated. The resulting solid was purified by silica-gel column chromatography (hexane/DCM volume ratio of 3:2 to 1:2). The solid was further purified by recrystallization from hexane/EtOAc to afford a white solid. Yield: 572 mg (63.5%). ^1^H NMR (400 MHz, CDCl_3_, ppm): δ 8.12 (t, 1H, *J* = 7.8 Hz, Ar-H), 7.51–7.40 (m, 3H, Ar-H). 7.06–6.95 (m, 4H, Ar-H), 2.58 (t, 2H, *J* = 7.4 Hz, -CH_2_-C=O-), 1.86–1.77 (m, 2H, CH_3_-CH_2_-), 1.07 (t, 3H, *J* = 7.6 Hz, CH_3_-). ^13^C{^1^H} NMR (100 MHz, CDCl_3_, ppm): δ 171.6, 162.2 (d, *J* = 260 Hz), 161.7 (d, *J* = 3.8 Hz), 159.6 (d, *J* = 250 Hz), 152.1 (d, *J* = 11 Hz), 151.1 (ddd, *J* = 249, 11, 4.8 Hz), 145.2 (td, *J* = 12, 4.5 Hz), 143.1 (d, *J* = 9.6 Hz), 138.3 (dt, *J* = 250, 15 Hz), 132.7, 130.7 (d, *J* = 3.8 Hz), 124.8, 123.9 (d, *J* = 12 Hz), 118.3 (d, *J* = 3.8 Hz), 117.7 (dd, *J* = 23, 3.4 Hz), 115.9 (d, *J* = 9.6 Hz), 110.7 (d, *J* = 26 Hz), 107.3–107.1 (m). 36.1, 18.4, 13.6. ^19^F NMR (376 MHz, CDCl_3_, ppm): δ −108.3 (1F, 2-fluorobenzoate), −115.2 (1F, 3-fluorophenolate), −133.8 (2F, 3,4,5-trifluorophenyl), −164.3 (1F, 3,4,5-trifluorophenyl). HRMS (FAB+) m/z: calcd for C_23_H_15_F_5_O_4_: 450.0891; found: 450.0894 (M), Error: +0.8 ppm. Anal. Calcd for C_23_H_15_F_5_O_4_: C 61.34, H 3.36%; found: C 61.42, H 3.18%.

4-11. ^1^H and ^13^C{^1^H} NMR spectra

**Figure S31** ^1^H NMR spectrum of **DIO-bpp-1** in CDCl_3_. 400 MHz.

**Figure S32** ^13^C{^1^H} NMR spectrum of **DIO-bpp-1** in CDCl_3_. 100 MHz.

**Figure S33** ^1^H NMR spectrum of **DIO-bpp-2** in CDCl_3_. 400 MHz.

**Figure S34** ^13^C{^1^H} NMR spectrum of **DIO-bpp-2** in CDCl_3_. 100 MHz.

**Figure S35** ^1^H NMR spectrum of **DIO-bpp-3** in CDCl_3_. 400 MHz.

**Figure S36** ^13^C{^1^H} NMR spectrum of **DIO-bpp-3** in CDCl_3_. 100 MHz.

**Figure S37** ^1^H NMR spectrum of **DIO-bpp-4** in CDCl_3_. 400 MHz.

**Figure S38** ^13^C{^1^H} NMR spectrum of **DIO-bpp-4** in CDCl_3_. 100 MHz.

**Figure S39** ^1^H NMR spectrum of **EST-bpp-1** in CDCl_3_. 400 MHz.

**Figure S40** ^13^C{^1^H} NMR spectrum of **EST-bpp-1** in CDCl_3_. 100 MHz.

**Figure S41** ^1^H NMR spectrum of **EST-bpp-2** in CDCl_3_. 400 MHz.

**Figure S42** ^13^C{^1^H} NMR spectrum of **EST-bpp-2** in CDCl_3_. 100 MHz.

**Figure S43** ^1^H NMR spectrum of **EST-bpp-3** in CDCl_3_. 400 MHz.

**Figure S44** ^13^C{^1^H} NMR spectrum of **EST-bpp-3** in CDCl_3_. 100 MHz.

**Figure S45** ^1^H NMR spectrum of **EST-bpp-4** in CDCl_3_. 400 MHz.

**Figure S46** ^13^C{^1^H} NMR spectrum of **EST-bpp-4** in CDCl_3_. 100 MHz.

**Figure S47** ^1^H NMR spectrum of **EST-1’** in CDCl_3_. 400 MHz.

**Figure S48** ^13^C{^1^H} NMR spectrum of **EST-1’** in CDCl_3_. 100 MHz.

**Figure S49** ^1^H NMR spectrum of **EST-bpp-5** in CDCl_3_. 400 MHz.

**Figure S50** ^13^C{^1^H} NMR spectrum of **EST-bpp-5** in CDCl_3_. 100 MHz.

**5. DFT calculation**

**Figure S51** Comparison of optimized structures of a) **DIO-bpp-n** (n = 1–4), b) **EST-bpp-n** (n = 1–4), c) **EST-1’**, and d) **EST-bpp-5** at top and side views. The direction of their dipole moment is also shown in the figures at top view. Energy values calculated are shown in the bottom. The optimized structures were obtained by DFT calculation using B3LYP/6-31+G(2d,p) basis set.

**Table S2** Comparison of energy and dipole moment (μ) values of DIO analogs. Optimized structures were obtained by DFT calculation with different basis sets with/without empirical descriptions.

**Table S3** Comparison of energy differences between isomeric analogs. The results of the optimization calculation are summarized in Table S1. Optimized structures Energy values in kJ mol^−1^ were obtained from by the corresponding energy values in Hartrees (1 Hartree = 2625.5 kJ mol^−1^).

**Figure S52** Profiles of energy differences of a-c) **EST-bpp-1** and d-f) **EST-bpp-3** against dihedral angle variation. a, d) φ_1_’. b, e) φ_3_. c, f) φ_4_. The optimized structures were calculated with the fix of dihedral angles and the energy differences were plotted.

**Figure S53** Different paring patterns of optimized structure of two single molecules of **EST-bpp-1**. a) (x, y, z). b) (x, -y, -z), c) (-x, y, -z), and d) (-x, -y, z) are 180° rotation arrangements of a) (x, y, z) around x-, y-, z-axes, respectively. e) (x, y, -z), f) (x, -y, z), g) (-x, y, z), and h) (-x, -y, -z) are inversion arrangements in the xy-plane of a) (x, y, z), b) (x, -y, -z), c) (-x, y, -z), and d) (-x, -y, z), respectively. By the combination of the arrangements of a) and b–h), 8 paring patterns can be produced. For Parallel-C and -D, DFT Calculations were not performed due to their large steric hinderance after paring.

**Figure S54** Comparison of optimized structures of parallel (a–c) and antiparallel pairs(d–i) of **EST-bpp-1** in different paring patterns. a) Parallel-A: (x, y, z) + (x, y, z). b) Parallel-B: (x, y, z) + (x, −y, −z). c) Parallel-B’: (x, y, z) + (x, −y, −z). d) Antiparallel-A: (x, y, z) + (−x, −y, z). e) Antiparallel-B: (x, y, z) + (−x, y, −z). f) Antiparallel-B’: (x, y, z) + (−x, y, −z). g) Antiparallel-C: (x, y, z) + (−x, −y, −z). h) Antiparallel-C’: (x, y, z) + (−x, −y, −z). i) Antiparallel-D: (x, y, z) + (−x, y, z). Parallel-B’, -C’, antiparallel-B’, and -C’ are in contact on opposite faces from the corresponding pairing patterns.

**Figure S55** Comparison of optimized structures of parallel (a–c) and antiparallel pairs(d–i) of **EST-bpp-3** in different paring patterns. a) Parallel-A: (x, y, z) + (x, y, z). b) Parallel-B: (x, y, z) + (x, −y, −z). c) Parallel-B’: (x, y, z) + (x, −y, −z). d) Antiparallel-A: (x, y, z) + (−x, −y, z). e) Antiparallel-B: (x, y, z) + (−x, y, −z). f) Antiparallel-B’: (x, y, z) + (−x, y, −z). g) Antiparallel-C: (x, y, z) + (−x, −y, −z). h) Antiparallel-C’: (x, y, z) + (−x, −y, −z). i) Antiparallel-D: (x, y, z) + (−x, y, z). Parallel-B’, -C’, antiparallel-B’, and -C’ are in contact on opposite faces from the corresponding pairing patterns.

**Table S4** Comparison of stabilization energy and dipole moment between single molecule and pairs in parallel and antiparallel arrangements in different pairing patterns. Top: **EST-bpp-1**. Bottom: **EST-bpp-3**. Optimized structures were determined by DFT calculation using B3LYP/6-31+G(d,p)/GD3BJ basis set. The paring patterns in parallel and antiparallel arrangements with the largest stabilization energy are marked red and blue, respectively.

^a^ Energy without the correction of counterpoise (CP) method. ^b^ denotes the dipole moment along to x-axis (longitudinal axis). ^C^ indicates the energy difference after pairing of two single molecules.

**Table S5** Comparison of stabilization energy between single molecule and pairs in parallel and antiparallel arrangements of **EST-bpp-1** and **EST-bpp-3**. Optimized structures were determined by DFT calculation using B3LYP/6-31+G(d,p)/GD3BJ basis set.

^a^ Energy corrected by counterpoise (CP) method. ^b^ denotes the dipole moment along to x-axis (longitudinal axis). ^c^ denotes basis set superposition effect which were calculated by CP method. ^d^ indicates the energy difference after pairing of two single molecules.

**References**

(1) Gaussian 16, Revision C.01, Frisch, M. J.; Trucks, G. W.; Schlegel, H. B.; Scuseria, G. E.; Robb, M. A.; Cheeseman, J. R.; Scalmani, G.; Barone, V.; Petersson, G. A.; Nakatsuji, H.; Li, X.; Caricato, M.; Marenich, A. V.; Bloino, J.; Janesko, B. G.; Gomperts, R.; Mennucci, B.; Hratchian, H. P.; Ortiz, J. V.; Izmaylov, A. F.; Sonnenberg, J. L.; Williams-Young, D.; Ding, F.; Lipparini, F.; Egidi, F.; Goings, J.; Peng, B.; Petrone, A.; Henderson, T.; Ranasinghe, D.; Zakrzewski, V. G.; Gao, J.; Rega, N.; Zheng, G.; Liang, W.; Hada, M.; Ehara, M.; Toyota, K.; Fukuda, R.; Hasegawa, J.; Ishida, M.; Nakajima, T.; Honda, Y.; Kitao, O.; Nakai, H.; Vreven, T.; Throssell, K.; Montgomery, J. A., Jr.; Peralta, J. E.; Ogliaro, F.; Bearpark, M. J.; Heyd, J. J.; Brothers, E. N.; Kudin, K. N.; Staroverov, V. N.; Keith, T. A.; Kobayashi, R.; Normand, J.; Raghavachari, K.; Rendell, A. P.; Burant, J. C.; Iyengar, S. S.; Tomasi, J.; Cossi, M.; Millam, J. M.; Klene, M.; Adamo, C.; Cammi, R.; Ochterski, J. W.; Martin, R. L.; Morokuma, K.; Farkas, O.; Foresman, J. B.; Fox, D. J. Gaussian, Inc., Wallingford CT, 2016.
